# Recent Advances and Methodological Considerations on Vaccine Candidates for Human Schistosomiasis

**DOI:** 10.3389/fitd.2021.719369

**Published:** 2021-08-26

**Authors:** Ursula Panzner, Jean-Louis Excler, Jerome H. Kim, Florian Marks, Darrick Carter, Afzal A. Siddiqui

**Affiliations:** 1 International Vaccine Institute, Seoul, South Korea,; 2 Swiss Tropical and Public Health Institute, Basel, Switzerland,; 3 University of Basel, Basel, Switzerland,; 4 Cambridge Institute of Therapeutic Immunology and Infectious Disease, University of Cambridge School of Clinical Medicine, Cambridge, United Kingdom,; 5 University of Antananarivo, Antananarivo, Madagascar,; 6 PAI Life Sciences Inc., Seattle, WA, United States,; 7 Center for Tropical Medicine and Infectious Diseases, Texas Tech University Health Sciences Center, Lubbock, TX, United States,; 8 Department of Internal Medicine, School of Medicine, Texas Tech University Health Sciences Center, Lubbock, TX, United States

**Keywords:** *Schistosoma*, schistosomiasis, vaccines, protective immunity, praziquantel, non-human primate model, controlled human infection model, efficacy endpoints

## Abstract

Schistosomiasis remains a neglected tropical disease of major public health concern with high levels of morbidity in various parts of the world. Although considerable efforts in implementing mass drug administration programs utilizing praziquantel have been deployed, schistosomiasis is still not contained. A vaccine may therefore be an essential part of multifaceted prevention control efforts. In the 1990s, a joint United Nations committee promoting parasite vaccines shortlisted promising candidates including for schistosomiasis discussed below. After examining the complexity of immune responses in human hosts infected with schistosomes, we review and discuss the antigen design and preclinical and clinical development of the four leading vaccine candidates: Sm-TSP-2 in Phase 1b/2b, Sm14 in Phase 2a/2b, Sm-p80 in Phase 1 preparation, and Sh28GST in Phase 3. Our assessment of currently leading vaccine candidates revealed some methodological issues that preclude a fair comparison between candidates and the rationale to advance in clinical development. These include (1) variability in animal models - in particular non-human primate studies - and predictive values of each for protection in humans; (2) lack of consensus on the assessment of parasitological and immunological parameters; (3) absence of reliable surrogate markers of protection; (4) lack of well-designed parasitological and immunological natural history studies in the context of mass drug administration with praziquantel. The controlled human infection model - while promising and unique - requires validation against efficacy outcomes in endemic settings. Further research is also needed on the impact of advanced adjuvants targeting specific parts of the innate immune system that may induce potent, protective and durable immune responses with the ultimate goal of achieving meaningful worm reduction.

## INTRODUCTION

### Disease Burden

Schistosomes are digenea trematodes within the clade of platyhelminths. Human pathogenic species include *Schistosoma haematobium* (Sh) and *S. mansoni* (Sm) occurring throughout Africa and the Middle East. The former is also found in Mediterranean Europe and the latter also in South America. *S. japonicum* (Sj) and *S. mekongi* occur in Central, East and South-East Asia (1–3). Schistosomiasis is a neglected tropical disease (4) that is estimated to affect more than 240 million people (5, 6). The 2016 Global Burden of Disease study suggested that of all diseases assessed, schistosomiasis revealed the most pronounced reduction in age-standardized years lived with disability (YLD) between 2006 and 2016. Low infection intensities with minimal clinical symptoms likely underestimate the true burden of infected individuals leading to a hypothetical correction of one egg-negative case for each egg-positive case and an adjusted 400 and 600 million infected people globally (7, 8). Initial infections occur in endemic settings with typical transmission patterns among young children two years of age with increasing infection intensities during the following 10 years of life, peaking among young adolescents and decreasing during adulthood. An estimated 60–80% schoolchildren and 20–40% adults have persistent infections (9, 10). Sh-HIV co-infections, especially among women suffering from genital mucosal inflammation and ulceration, and Sm-*Plasmodium* spp. co-infections are considered important contributors to the HIV and malaria epidemics in Africa (11–14). Genital schistosomiasis caused by Sh is a common gynecologic condition affecting an estimated 40 million African women (15).

### Parasite Pathogenicity

Definitive mammalian hosts acquire infection *via* skin penetration during contact with fresh water infested with infectious larval cercariae released from intermediate species-specific snail hosts. Cercariae transform to juvenile schistosomes or schistosomula before entering the vascular system, where they migrate to their venous destination to mature and mate with their sexual counterparts (16). Adult worms spend much of their lives *in copula* and are capable of existing in immunocompetent hosts for decades. The adult worms migrate to the mesenteric venules of the intestine, i.e. Sm and Sj, or the venous plexus of the bladder, i.e. Sh, where females residing in the gynaecophoric canal of males produce hundreds of eggs daily that are subsequently fertilized by males. Ova released *via* feces for Sm and Sj or urine for Sh continue their lifecycle upon hatching in fresh water as miracidia and multiplication by asexual replication in snail hosts prior to transmission to definitive hosts.

Clinical disease in humans can be divided into acute and chronic manifestations. Acute schistosomiasis is seen among individuals without previous parasitic exposure and presents as debilitating febrile illness at six to eight weeks post infection. Chronic schistosomiasis is caused by inflammatory, immunopathological host responses and organ damage due to bleeding, scarring, and formation of granulomata and fibrosis around eggs retained in tissues. Schistosomiasis clinical features are parasite species-dependent with intestinal, hepatic and urinary pathology including malignancies, e.g., squamous cell carcinomas in the bladder or sandy patches in the female genital areas. It may affect other organs such as the central nervous system through ectopic ova deposition, and lead to cognitive and physical impairment - especially among children (7, 17, 18).

Schistosomes co-exist in immunocompetent hosts for decades since they adapt, modulate, and evade cellular and humoral immune defense mechanisms though with parasite species-specific differences (9, 19). Their pivotal component for switching from a young immune-sensitive to an adult immune-refractory state is the tegument. It is a syncytial matrix of fused cells overlaid by a lipoidal membranous bilayer on the parasite outer surface, and essential for metabolic processes, movement, and external interchange (16, 20–22). Tegumental mechanisms to evade host defense components include biophysical membrane adaptations with rapid turnover, and alterations or masking of surface antigens and immunomodulatory molecules (21).

### Host Immunity

Upon infection in humans, the immune system is confronted with multiple moieties exposed during the various lifecycle stages. Intact adult worms are impervious to immune attacks, whereas the migrating skin- and especially lung-stage schistosomula are targets of the cellular and humoral host defense (9, 10, 23). Generally, hosts react in the following general manner against schistosomal infections: (i) development of age-dependent partial protective immunity to reinfection from repeated adult worm death that releases immunologically active molecules that induce adaptive immunity and (ii) initiation of immunopathogenic and/or immunoregulatory mechanism against parasitic antigens released from tissue-trapped ova (7, 17, 24, 25). In endemic settings, immune responses and disease severity are influenced by multiple host factors, e.g., infection intensity, cumulative treatment history, co-infection status, genetics/genetic pre-disposition, and *in utero* transplacental and postnatal oral antigen sensitization resulting in phenotypically similar maternal and fetal responses (18, 26). Interestingly, newborns of mothers with schistosomiasis have a pre-existing anti-inflammatory T-helper 2 (Th2) response, IgM and/or IgE anti-schistosome antibodies, and cord blood mononuclear cells proliferating with anti-egg antibodies that induce altered regulated responses to infections in these children (10, 18, 26).

The acute disease phase is characterized by pro-inflammatory CD4+ T-helper 1 (Th1) or CD4+ Th1/T-helper 17 (Th17) responses against migrating schistosomula with elevated production of tumor necrosis factor alpha (TNF-α) and interferon gamma (IFN-γ) triggering innate phagocytic antigen-presenting and epithelial cells to release larvicidal molecules and other cytokines (18, 23, 27–30). For instance, interleukin (IL)-17-activated neutrophils release neutrophilic extracellular traps (NETs) to sequester schistosomula in the bloodstream; IL-10-regulated IL-12 is essential for Th1-cell differentiation (23, 31). Regulatory CD4+ T-cells (Treg) provide an essential regulatory arm to stabilize the immune response and limit immunopathology. Tregs and cytokine decoy receptors serve to limit the extent of immune-mediated pathology during schistosomiasis (32). However, in Sh-infected individuals Treg proportions rise significantly with increasing infection in younger age groups. In contrast, Treg were negatively correlated to infection intensity in older age groups (32, 33). The chronic disease stage is defined by anti-inflammatory CD4+ Th2 responses against egg epitopes with antigen-presenting cells, members of the B7 superfamily, and cytokines to downregulate the pro-inflammatory response (18). IL-10, which has a role in T-cell regulation with IL-4, prevents damages from Th1/Th2-mediated pathologies, and polarizes Th1/Th2 responses to enhance resistance to pathogens, and mitigate disease severity, thus improving host survival (27, 28, 34).

Extreme polarization is detrimental; the “happy valley” hypothesis assumes best anti-parasite protection at either the Th1- or the Th2-pole, and least protection at a well-mixed Th1/Th2 balance where parasites seem “happiest” (27). IL-4 activates phagocytic antigen-presenting and cytokine-producing leukocytes and interacts with other cells presenting antigens in endemic populations impacting vaccine efficacy and disease susceptibility. Whether persistent schistosomiasis affects HIV susceptibility or both agents interact is uncertain though viral replication is higher when Th2-cells are present (18). Though Th2-cells stimulate natural but only partial non-sterile resistance to reinfection, they contribute long-term to disease chronicity with granulomatous-fibrotic formations mediated by IL-4, IL-5 and IL-13, and STAT6 (signal transducer and activator of transcription 6) pathways (32).

The B-cell contribution to parasite clearance and prevention of disease severity is not well understood. IgE and IgA antibodies confer protective immunity with resistance to reinfection, and larval killing through antibody-dependent cell-mediated cytotoxicity (ADCC) along with blood effector cells (27, 35–37). However, IgE antibodies develop slowly due to long-lived schistosomes and antigen-exposure occurring only during parasitic death. In contrast, IgG4, IgG2 and IgM against parasitic carbohydrate epitopes are associated with susceptibility to reinfection and disease severity along with mediators, e.g., IFN-γ, Treg and IL-10, thus blocking protective antibodies (10, 35–37). Whether resistance to schistosomiasis is due to a balanced mix of antibodies or to the presence or absence of a particular antibody is unclear (35, 36). Vaccine-induced hypersensitivity due to pre-existing IgE from past infections are seen in other worms, e.g., for the Brazilian APS-based hookworm vaccine in its Phase I trial (38, 39).

### Prevention and Treatment

Preventive measures are multifaceted and include avoiding contact with infectious water, improved access to uncontaminated water, improved hygiene and sanitation, elimination of intermediate freshwater snail hosts, and provision of health education to high-risk populations, especially in settings with dam construction, irrigation systems, and internally/externally displaced persons (refugee camps) (40, 41). The drug of choice remains praziquantel (PZQ) (42). It is efficacious against adult worms by changing irreversibly the tegumental permeability and stability but works poorly against schistosome larva (9). Its full efficacy is impacted by host factors, e.g., immune defense, infection intensity, exposure history, gut microbiota, and physiological disposition and bioavailability, and parasite factors, e.g., localization in bloodstream leading to damage or recovery (9, 19, 20, 24, 42–45). Anti-worm rather than anti-egg antibodies are reported to increase after PZQ treatment. Initially, IgG4 leads to higher susceptibility to re-infection due to IgE blocking but a decrease in susceptibility with regular repeated treatment is seen due to decreased IgG4 levels (46, 47). The immunoregulatory role of PZQ is driven by the promotion of CD4+ T-cells and differentiation of Type 1 regulatory cells to maintain immune hemostasis and effector cytokine secretion - in particular IL-4, IL-5 and IL-10 (10, 45). However, since PZQ cannot prevent reinfection, regular repeated large-scale mass drug administration (MDA) is needed in endemic settings, which requires large infrastructure and financial investment. About 105 million people received preventive treatment in 2019, though at least 237 million people were in need (1, 44). The ability of PZQ to cure adult worm infection and reduce egg excretion is important, but concern over the potential of emerging resistance has arisen because of its exclusive and extensive use over decades (7–9, 39, 42, 48, 49).

## ADVANCED VACCINE CANDIDATES

Given the limitations of MDA alone, a hypothesis is that complementing a comprehensive approach with a safe and effective vaccine would more likely be successful in dramatically reducing the incidence of schistosomiasis (7). Current research builds largely on the radiation-attenuated cercarial vaccine considered as gold standard in terms of immunological response in experimental models, but difficult to be translated for human use. Single percutaneous administration yields 60–70% protection against subsequent challenge infection starting about two weeks post-immunization and persisting for about 15 weeks in various animal species and for several *Schistosoma* species (31, 50).

Preferred product characteristics for a schistosomiasis vaccine have been proposed (7, 8). Since schistosomes do not replicate in their definitive host, a prophylactic vaccine against schistosomiasis should ultimately result in reduction of disease manifestations as well as blocking transmission, thus reducing force of transmission (7, 8). The goal is non-sterilizing immunity with a long-term decline in tissue eggs and egg excretion - preferably through killing of female worms - while preserving concomitant natural immunity induced by non-pathogenic male worms (8, 51–53). A candidate vaccine should be co-administrable with PZQ in MDA programs and with existing national vaccination programs. In the 1990s, a joint UNICEF, UNDP, World Bank, WHO-TDR program promoting vaccines against parasitic diseases shortlisted some promising candidates; only few have progressed to pre-clinical and clinical stages: Sm-TSP-2 which is in Phase 1b/2b, Sm14 in Phase 2a/2b, Sm-p80 which is being submitted to the US FDA for Phase 1 clinical trial approval, and Sh28GST which has completed Phase 3 trials (51, 54, 55). We present findings from the literature and clinical trials registered at https://clinicaltrials.gov. For each of the four leading vaccine candidates, we discuss the antigen, and preclinical and clinical studies as applicable. Details on antigens, formulations, study designs and findings are summarized in Supplementary Tables S1–S4.

### *S. mansoni* Tetraspanin: Sm-TSP-2/Sm-TSP-2/Al^®^

**FIGURE 1 |:**
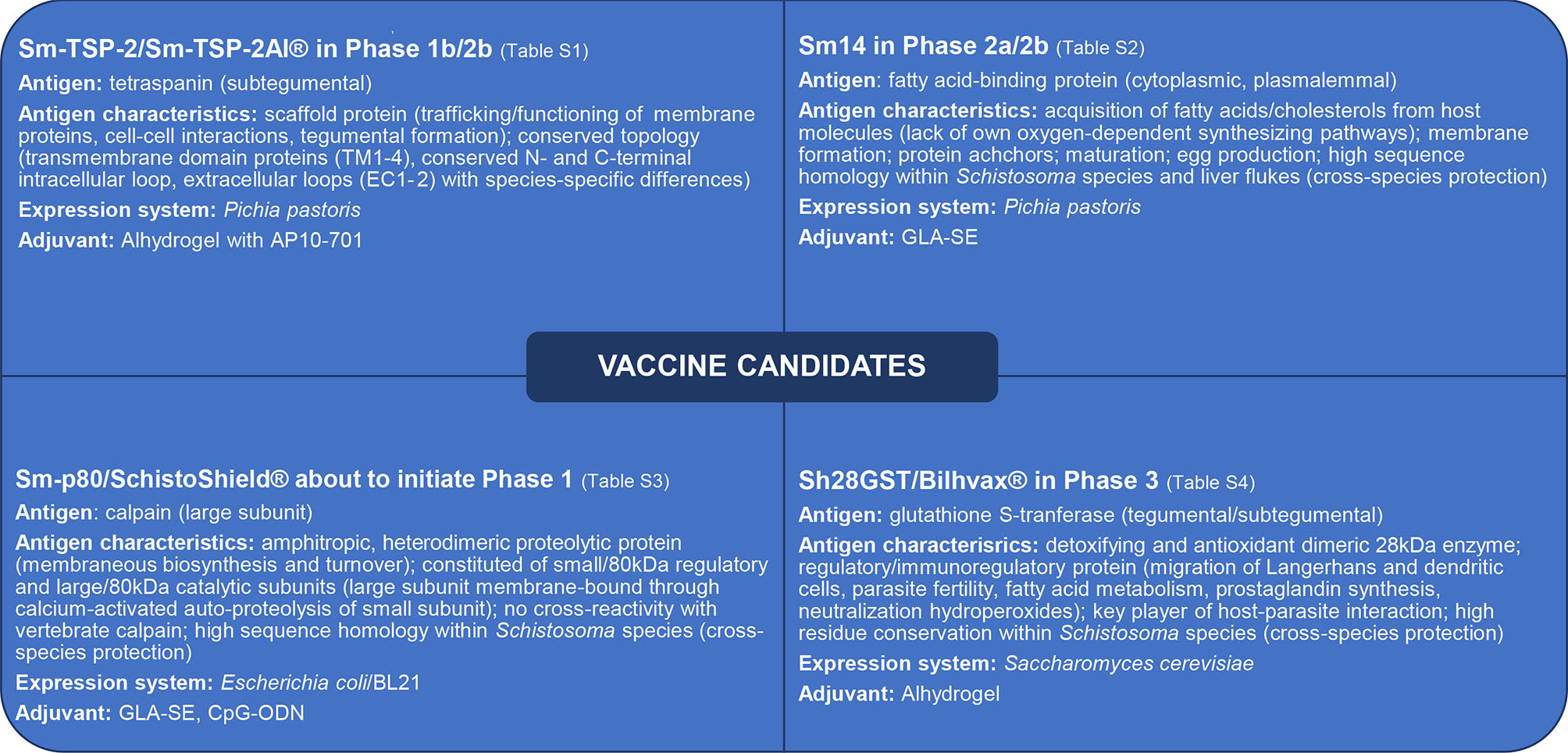
Antigen characteristics of advanced schistosomiasis vaccine candidates which progressed to pre-clinical and clinical development (details in Supplementary Tables S1–S4). Sm, Schistosoma mansoni; Sh, Schistosoma haematobium; TSP, tetraspanin; GST, glutathione S-transferase; cGMP, current Good Manufacturing Practice; AP10–701, glucopyranosyl lipid A (aqueous formulation); GLA-SE, glucopyranosyl lipid A in stable emulsion (TLR4 agonist); CpG-ODN, oligodeoxynucleotides with unmethylated CpG dinucleotides (TLR9 agonist).

The characteristics of tetraspanin (TSP) are summarized in Figure 1 (56–61); detailed pre-clinical and clinical investigations of Sm-TSP-2 are presented in Supplementary Table S1.

TSPs are scaffold proteins regulating the trafficking, functioning of membrane proteins, cell-cell interactions, and tegumental formation; they share a topology of an intracellular N- and C-terminal loop, and extracellular loops EC1 and EC2 with species-specific phylogenetic differences stimulating differential protection (56–60). TSP’s species-specific genetic polymorphism - especially within the EC2 external loop - and subclass diversity impact its host range and suitability as a target of protective immunity in addition to cross-species protection (62). Gene sequences of the EC2-domain from Kenyan Sm male worms revealed the greatest degree of polymorphism found in *Schistosoma* spp (58). The sub-tegumental Sm-TSP-2 of newly transformed schistosomula is an easy target of protective immunity and uniquely recognized by IgG1 and IgG3 antibodies from individuals with acquired resistance to schistosomiasis compared to chronically-infected subjects. Krautz-Peterson et al. showed that a limited number of dominant conformational epitopes on five major tegumental surface membrane proteins, i.e., Sm-TSP-2, Sm23, Sm29, SmLy6B and SmLy6F, are primary targets of serum antibodies from mice, rats and humans infected with Sm. However, neither infecting schistosomula nor mature adult schistosomes are substantively impacted by the robust circulating antibodies specific to these antigens (62). By contrast, Th1 responses to these antigens can be harmful to schistosomes and are responsible for the reduction in parasite load following vaccination (63, 64).

Screening sera of individuals from endemic areas of the state of Minas Gerais, Brazil revealed higher anti-TSP-2 IgG1 and IgG3 antibodies among putatively resistant individuals, though no anti-TSP-1 antibodies were found. However, among chronically-infected individuals, all IgG subclasses and IgE were detected against soluble egg and worm antigens (65). Injection of mice with schistosomula after electroporation with *Sm-TSP-1* and *Sm-TSP-2* dsRNAs resulted in 61% and 83% reductions in the numbers of parasites recovered from the mesenteric veins four weeks later when compared to dsRNA-treated controls. These results imply that TSPs play important structural roles impacting tegument development, maturation, or stability (61).

TSP orthologs in Sh and Sj with >90% sequence homology indicate potential cross-species protection (66, 67). Protection based on subclass diversity with differential expression of transcripts across the parasitic lifecycle stages and higher expression in female than male worms was investigated within Sj-TSP-2. Immunizing mice with Sj-TSP-2 subclass C or recombinant Sj-TSP-2 subclasses A-G resulted in no protection (56, 66); however, administering Sj-TSP-2 subclass E or D adjuvanted with or without complete Freund’s adjuvant (CFA) or incomplete Freund’s adjuvant (IFA) resulted in reductions of 54% hepatic and 69% fecal eggs, and 53% hepatic and 52% fecal eggs, respectively (56, 66, 68). Strong total IgG (IgG1 and IgG2a) antibody responses with weak IgA, IgE and IgM levels were detected, indicating an overall marked Th1/Th2 response (65).

Variations in the Sj-TSP-2 EC2 sequence may alter the affinity or avidity to hosts’ immune responses and stimulate different levels of protective efficacy. Experiments in mice with Sm-TSP-2 encoding EC2 or Sm-TSP-2/5B chimera (hookworm vaccine candidate) co-administered with CpG/alum reduced worm burden by 25–27% and 54–58%, and liver eggs by 20–27% and 48–56%, respectively, and induced higher titers of total IgG, IgG1 and IgG2 antibodies in the Sm-TSP-2/5B group (65, 69). Analysis of sera of chronically-infected individuals from Sm and hookworm endemic Minas Gerais, Brazil, did not reveal Sm-TSP-2-specific (and Na-APR-1/5B) IgE antibodies despite high IgE titers to crude schistosome soluble egg antigen.

Additional pre-clinical experiments combining Sm-TSP-2 with Sm29 adjuvanted with CFA/ICA conferred increased protective efficacy (63). Similar to the aforementioned studies, individuals from endemic areas in Brazil showed higher IgG against Sm-TSP-2/Sm29 in naturally-resistant individuals, suggesting that a multivalent vaccine might be better recognized by individuals with a resistant phenotype (63). Murine experiments with Sm-TSP-2/Sm29 vaccination resulted in higher reductions of worm burden and granulomata, with elicitation of total IgG, IgG1 and IgG2a antibodies with IFN-γ and TNFα cytokine production across groups, and a Th1 response with increasing antibody titers following additional booster immunizations (63). Mice immunized with DNA-based Sm29, Sm-TSP-2, Sm29 N/C-terminus/Sm-TSP-2 chimera or Sm29+Sm-TSP-2 had worm reductions of 17–22%, 22%, 31–32% or 24–32% and decreases in hepatic granuloma of 28%, 30%, 37% and 26% respectively (70). High total IgG to N/C-terminus/SmTSP-2 chimera and Sm29+rSm-TSP-2 along with high production of IFN-γ and TNF-α -indicative of a Th1-biased profile - were detected following cercarial challenge. DNA-based immunization resulted in a lower protection rate compared to recombinant protein formulations. A chimeric Sm multi-epitope (Sm14, Sm21.7, Sm23, Sm29, Smp80, Smcb, and Sm-tsp-2), subunit vaccine formulated with a novel TLR4 agonist induced humoral and cellular immune responses suggestive of its potential as a prophylactic or therapeutic vaccine (71). Taken together, these findings justified taking Sm-TSP-2 (made in *Pichia pastoris*) forward in clinical trials.

Sm-TSP-2/Alhydrogel (Sm-TSP-2/Al^®^) with or without Glucopyranosyl Lipid Adjuvant in an Aqueous Formulation (“GLA-AF”) was first tested in a Phase 1 dose-escalation trial among healthy adults from a Sm non-endemic area (NCT02337855) (49, 72). The vaccine was well tolerated with only mild local and systemic reactogenicity and no vaccine-related serious adverse events reported. The proportion of vaccinees responding 14 days post vaccination was 30%, 50%, and 89% for 10μg, 30μg and 100μg antigen doses, respectively, suggesting a dose-dependent response. IgG antibodies peaked at day 127 post vaccination in the 30μg and 100μg cohorts, but were not detectable in the 10μg cohort, and decreased by day 293 across cohorts. Magnitude and longevity of responses to Sm-TSP-2/Al^®^ with GLA/AF (AP10–701) were assessed in a subsequent dose-escalation Phase Ib trial in healthy adults from a Sm endemic Brazilian setting (NCT03110757), but the findings are yet to be published (73). Safety, immunogenicity, efficacy and cross-species protection against Sh of Sm-TSP-2/Al^®^ with AP10–701 are being investigated in Phase I and Phase IIb trials among healthy adults from a Sm endemic setting in Uganda (NCT03910972) (74).

### *S. mansoni* Fatty Acid-Binding Protein: Sm14

The characteristics of FABP are provided in Figure 1 (75–79); pre-clinical and clinical investigations of Sm14 are in Supplementary Table S2.

FABPs are expressed in all parasitic life cycle stages. Cytoplasmic FABPs allow schistosomes to acquire fatty acids and cholesterol from the host due to a lack of schistosome oxygen-dependent synthetic pathways essential for membrane formation, protein anchoring, maturation, and egg production (75–79). Sm FABP shares 91%, 45%, 39% and 49% sequence homology with FABPs of Sj, *Echinococcus granulosus, Fasciola hepatica*, and *Clonorchis sinensis*, respectively; phylogenetic proximity may suggest potential for cross-species protection (80–83).

Sm14-vaccinated mice exhibited a 41% reduction in worms compared to unvaccinated controls; immunizing outbred rodents with Sm14 formulated with or without CFA reduced Sm worms by 66%–89% and *Fasciola hepatica* metacercariae by 100% (84–87). Administering adjuvanted Sm14 to ungulates resulted in high cross-species protection against *Fasciola hepatica* parasites and liver damage due to T-lymphocyte infiltration, with strong IgM and IgG responses (88–90) suggesting Sm14 as a vaccine candidate with cross-species potential (91, 92). Goats immunized with synthetic Sm14 adjuvanted with RIBI/Al(OH)_3_ developed cross-species cellular responses and strong humoral protection also against *Fasciola hepatica* with declines in liver and gallbladder worms of 46%, and a reduction of gross liver damage of 55% due to low infiltration of CD2+, CD4+ and CD8+ T lymphocytes (87–90). Early murine experiments showed worm reductions ranging from 20–67%, and strong Th1-predominant responses without impacting granulomatous-fibrotic reactions (92–100). Further rodents immunized with Sm14 with CFA or pRSET-His-rSm14, showed 72% protection and prevention of parasite maturation and hepatic tissue damage by 100% (79, 84–87, 101).

Naturally resistant individuals in endemic areas demonstrate a Th1 immune response to Sm14. Assessment of cell-mediated responses among subjects from endemic areas in Brazil using synthetic multi-epitope peptides of Sm14 (1–18, 32–48, and 53–69) indicated that Sm14 epitopes 32–48 and 53–69 were recognized by the majority of individuals tested and preferentially by resistant patients compared to non-infected, infected and susceptible subjects. A marked Th1 immune response profile corresponded with decreased granulomatous fibrotic pathology (99, 102–105). Mixtures of synthetic peptides of Sm14 and paramyosin with CFA/IFA induced a Th1/Th2 immune response with noticeable reductions of worms, intestinal eggs, and hepatic granuloma suggesting that multi-epitope-based vaccines increase the frequency of responders in genetically distinct populations. Multi-epitope and multi-valent (Sm14 and Sm29) adjuvanted vaccine candidates elicited Th2-dominant protection and a decline in granulomatous-fibrotic pathologies in different animal models (102–105). Sm14 antigens from different expression systems, e.g., *Mycobacterium* spp., *Clostridium* spp., and *Salmonella* spp., were of moderate efficacy (101, 106–110). Subsequent optimization of expression and fusion systems and adjuvant formulations elicited strong total IgG and IgG subclass responses with induction of cells positive for IFN-γ, TNF-α and other cytokines associated with reductions in worms, hepatic granuloma, and egg counts (92–101, 106–110). Similarly, bivalent constructs (Sm14/Sm28 GST expressed in *Escherichia coli*, Sm14/Sm29, and FSm14/29 adjuvanted with poly(I:C), administered in murine models resulted in declines in adult worms, hepatic and intestinal eggs, and hepatic granuloma size and frequency, and elicitation of Th1 immune responses (87, 103–106, 110).

Sm14 expressed in *Pichia pastoris* and adjuvanted with GLA in a stable emulsion (GLA-SE) advanced into a safety and immunogenicity Phase 1 clinical trial among healthy adults from a non-endemic Brazilian area (NCT01154049) (111–113). The vaccine given intramuscularly at weeks 0, 4 and 8 was safe and well tolerated, and no vaccine-related serious adverse event was reported. Sm14-specific total IgG and IgG1–4, were induced from day 30 until day 90 together with Th-1 and Th-2 cytokines; no IgE were detected suggestive of reduced risk of vaccine-induced hypersensitivity. Safety and immunogenicity of Sm14/GLA-SE were assessed in a Phase 2a trial among adults with history of Sm and/or Sh infections from the hyperendemic Senegal River Basin, Senegal (114). Adults received one dose of PZQ three weeks prior to vaccination (NCT03041766) (113, 114). Preliminary results suggest that Sm14/GLA-SE was safe and well tolerated and resulted in 92% seroconversion after the second booster dose. Sm14-specific antibody titers were detected up to 12 months after initial immunization (113). A Phase 2b 3-arm self-contained open-label controlled randomized trial is ongoing among healthy and Sm- and/or Sh-infected schoolchildren from the Senegal River Basin receiving a single dose of PZQ prior vaccination to further assess the safety and immunogenicity of Sm14/GLA-SE (NCT03799510) (113, 115).

### *S. mansoni* Large-Subunit Calpain: Sm-p80/SchistoShield^®^

The characteristics of calpain are summarized in Figure 1 (116–125); detailed pre-clinical results using Sm-p80 are in Supplementary Table S3.

Calpain is a proteolytic protein for membranous biosynthesis consisting of a catalytic and a regulatory subunit. The large subunit, Sm-p80, changes to a membrane-bound status through calcium-activated auto-proteolysis by the small subunit (124, 125). It shows no immunological cross-reactivity with calpains in vertebrates; Sm-p80 is expressed in all schistosome lifecycle stages though with a 2.5-fold higher expression in females compared to male adult worms, and plays a pivotal role in membranous biosynthesis and turnover (116–123). Sm-p80 induced cross-species protection against Sh with reductions of 48% worms, and 66% and 63% hepatic and intestine eggs in Syrian hamsters, respectively, and reductions of 25% worms, and 64% urinary bladder, 40% fecal and 53% urinary eggs in *Papio anubis* baboons with robust immune responses indicative of balanced Th1/Th17 immunity though slightly biased to a Th2 response (125, 126). Cross-species protection was also shown against Sj using GLA-SE-adjuvanted Sm-p80, and against Sh using a Sm-p80-VR1020 DNA prime and CpG-ODN-adjuvanted protein boost approach with reductions of 47% of worms and 5% of hepatic eggs in mice, and reductions of 27% worms but no effects on hepatic, intestinal and urinary eggs in hamsters, respectively, and a balanced Th1/Th2 immune response (122, 126).

Sm-p80 expressed in baculovirus and adjuvanted with CFA resulted in 67% worm reduction in immunized mice challenged with Sm (124). Neither subcutaneous nor intranasal delivery of recombinant vaccinia virus-expressed protein-based Sm-p80 decreased adult worms, while DNA immunization *via* gene gun provided 60% protection with a Th1-based immune response against cercarial challenge (127). Sm-p80 DNA administered intramuscularly with IL-2 or IL-12 to mice at weeks 0, 4 and 8 followed by cercarial challenge resulted in 57% and 45% decreases in adult worms, respectively. Total IgG, IgG2a and IgG2b antibodies were enhanced in the presence of either interleukin indicative of a Th1 response, but no IgA, IgE and IgM antibodies were detected, which is a hallmark of Sm-p80 (52). The same DNA construct co-administered with the granulocyte-macrophage colony-stimulating factor (GM-CSF) or IL-4 conferred similar worm reductions of 44% and 42% compared to IL-2 or IL-12, respectively; Sm-p80-DNA given with GM-CSF or IL-4 resulted in the augmentation of both Th1 and Th2 responses, but no IgA, IgE and IgM antibodies (116). Subcutaneous immunization of mice with Sm-p80 DNA alone or adjuvanted with GM-CSF, IL-4, IL-12, or IL-2 followed by Sm cercarial challenge confirmed protective effects with decreased worm burden as in earlier experiments. Mouse splenocytes showed high *in vitro* proliferation indicative of a protective Th1 immune response (128). Increasing the frequency of DNA boosts resulted in 59% and 84% decrease of worm and egg burdens, respectively, with distinct total IgG, IgG1, IgG2a, IgG2b, IgG3 titers mediated by IFN-γ and IL-2 even without added adjuvants (129).

Vaccinating mice in a Sm-p80 DNA prime and protein boost *versus* Sm-p80 alone co-administered with Resiquimod (R848, TLR7/8 agonist) resulted in 49% and 50% worm as well as 30% and 16% egg reductions, respectively. In contrast, the same prime-boost regimen *versus* recombinant protein alone co-administered with CpG-ODN conferred decreases of 57% and 70% in adult worms, and 71% (65% anti-fecundity) and 75% (77% anti-fecundity) reduction in egg counts, respectively (30). The use of R848 resulted in high titers of IgM, IgA, total IgG and IgG1, IgG2a, IgG2b, and IgG3 together with pro-inflammatory cytokines in addition to IL-15 and IL-16 indicating a Th1 type immune response (130) - similar to the immune response seen in the CpG-ODN experiment though titers were higher with Sm-p80 alone. Both approaches elicited strong mixed Th1/Th17 and Th2 biased cellular responses (30).

*Papio anubis* baboons which develop a schistosomiasis syndrome similar to humans and are natural hosts for various *Schistosoma* species were vaccinated intramuscularly with 500μg Sm-p80 DNA alone or IL-2-adjuvanted followed by three boosts at 4-week intervals (8, 131). Immunized animals exhibited a Th1 response with 21–34% complement-dependent killing of schistosomula. A proof-of-concept study in non-human primates assessed the safety and prophylactic anti-fecundity potential of non-adjuvanted DNA immunization followed by a subcutaneous challenge with 1,000 Sm cercariae. The vaccine was well tolerated and showed 38% reduction in worms (30% males and 14% females), 50% reduction of paired worms, 6% reduction in immature worms, and 32% reduction hepatic and intestinal eggs, and a mixed Th1/Th2 response with Th1 dominance (118, 132–134).

Sm-p80 DNA cloned in vector VR1020 administered to mice resulted in a decrease in 47% adult worm and high total IgG and IgG2b titers following cercarial challenge indicative of a Th1/Th17 immune response (118). Baboons immunized with Sm-p80-VR1020 showed similar reductions in adult worms of 46%, and of hepatic and intestinal eggs of 28%, and a mixed Th1/Th2 response (133). The co-administration of Sm-p80-VR1020 or Sm-p80 protein with alum in mice saw reductions of 61% and 55% adult worms and 23% and 21% eggs counts, respectively, coupled with a robust Th1/Th2 response with strong IgM, total IgG, IgG1, IgG2 and IgG3 levels, and upregulation of pro-/anti-inflammatory cytokines affecting Th17 and Treg functions (134). Immunization of non-human primates with Sm-p80-VR1020 followed by two boosts 4 weeks apart with Sm-p80 protein adjuvanted with CpG-ODN or R848 resulted in adult worm declines of 47% and 38% *versus* 58% and 52% with protein alone, respectively. Total IgG, IgG1, IgG2 and IgA antibodies coupled with IFN-γ and IL-2 expression in peripheral blood mononuclear cells, splenocytes and lymph node cells, but no IgG3 and IgG4 were elicited in either group, indicative of a mixed/balanced Th1/Th17 and Th2 response (39).

Human correlate studies revealed Sm-p80 reactivity with immunoglobulin G in human serum samples from schistosome-infected individuals. In addition, a complete lack of prevailing Sm-p80–specific immunoglobulin E in high-risk or infected populations was observed, thus minimizing the risk of hypersensitivity reaction following vaccination with Sm-p80 in humans (39). This study provided the proof-of-concept to move Sm-p80 forward into further translational development leading to human clinical trials.

In order to dissect the role(s) of antibodies in Sm-p80 mediated protection, pooled sera from mice immunized with Sm-p80-DNA or purified IgG from baboons immunized with Sm-p80-DNA were transferred intravenously into naïve mice, respectively, prior to challenge with cercariae. Passive transfer of sera or purified IgG antibodies from mice and baboons vaccinated with Sm-p80 DNA into naïve mice with subsequent cercarial challenge resulted in decreases in adult worms, hepatic eggs and egg hatching from tissues, suggesting that antibodies play a significant role in Sm-p80-mediated protection (135). Sera from immunized baboons were able to kill a significant proportion of schistosomula in a complement-dependent manner (135, 136). Naïve *versus* antibody gene locus knockout mice immunized with Sm-p80 with CpG-ODN showed reductions of 63% and 18% adult worms, and 47% and 36% eggs, respectively; this decrease in protection demonstrates that antibodies in addition to complement are an essential component- but not the only one - of Sm-p80-mediated protection. Immunization of wild-type and complement-3-deficient mice with Sm-p80 with CpG-ODN showed that complement plays a minimal protective role *in vitro* though it may impact the development of egg-induced pathology. Worms were reduced by 53% and 34%, respectively, while 15 to 25-fold lower levels of trapped hepatic and intestinal tissue eggs were seen in complement-3-deficient than wild-type mice (119). ADCC protection using lung lavage and lung cells was assessed in mice vaccinated with Sm-p80 with CpG-ODN (137). High levels of larvicidal killing and cellular attachments of macrophages, lymphocytes, and endothelial cells activated by cytokines were found suggesting that at least in mice lung cells play pivotal roles in Sm-p80-mediated immunity to schistosomiasis. Sm-p80-specific IgG antibodies were detected up to 60 weeks in mice and 5–8 years in baboons, including maternal co-protection through placental transfer, colostrum or lactation after administration of Sm-p80 with GLA-SE and Sm-p80-DNA with IL-2, respectively (137, 138).

Immunization of chronically infected baboons with Sm-p80 adjuvanted with GLA-SE resulted in declines of 36% adult worms, 54% tissue eggs, and 33% fecal eggs; immunizing baboons with a Sm-p80-VR1020 DNA prime and Sm-p80 protein boost adjuvanted with alum or CpG-ODN led to reductions of 10% worms, 10% tissue eggs and 15% fecal eggs, and of 23% worms, 57% tissue eggs and 13% fecal eggs, respectively (139). Total IgG and IgM were detected in all groups, but the expression of IgA and IgG subclasses, and cytokines differed; balanced Th1/Th17 and weak Th2 profiles were potent to kill established worms, and to reduce egg retention and expulsion (133, 139, 140). Immunofluorescence studies on Sm-p80 show it is highly expressed on eggs while still in the uterus of females, which may suggest that eggs may be damaged by Sm-p80-specific antibodies through tegument permeation. Correspondingly, infected baboons immunized with Sm-p80 with GLA-SE had a 68% reduction in liver eggs with 86% decline in the hatching rate (141). Following vaccination of baboons with Sm-p80 with GLA-SE, egg levels were reduced in liver (91%), small intestine (87%), and large intestine (91%). Female adult worms and egg hatching into miracidia decreased by 93% and 82%, respectively (123, 126). The retention of some non-pathogenic male worms of stunted growth and shorter life spans due to lack of pairing in the absence of female worms may be of benefit in recurrent boosting of the natural immunity and resistance to schistosomiasis. Livers had soft, smooth texture with fewer, smaller granulomas and necrosis surrounding trapped eggs compared to control animals (123).

Sm-p80-based vaccine efficacy was evaluated in a baboon model of infection and disease. The efficacy study aimed to replicate the scenario of implementing Sm-p80 in endemic settings subsequent to drug treatment of infected individuals. Sm-p80-based vaccination reduced hepatic- (38%), small intestine- (72.2%) and large intestine-egg burdens (49.4%). Sm-p80 also reduced hatching rates by 60.4%, 48.6%, and 82.3%, respectively (142). Observed declines in egg maturation and hatching rates were supported by immunofluorescence and confocal microscopy revealing unique differences in Sm-p80 expression in worms of both sexes and matured eggs. Immunizing baboons resulted in 64.5% reduction of the urine schistosome circulating anodic antigen, a parameter that reflects worm numbers and health status of infected hosts. Total IgG titers were unchanged during trickle infection but increased following PZQ treatment and vaccination. Transcriptomes of peripheral blood mononuclear cells, and secondary lymphoid tissues among vaccinated baboons exhibited induction, activation and proliferation of innate and adaptive humoral and cellular elements, including memory immune responses (142). Phase 1 clinical trials are proceeding now in US adults (first-in-human) followed by dose-escalation among African adults with future age-de-escalation to children with SchistoShield^®^ (Sm-p80 expressed in *E. coli* and adjuvanted using GLA-SE).

### *S. haematobium* Glutathione S-Transferase: Sh28GST (Bilharvax^®^)

The characteristics of GST are summarized in Figure 1 (143–151); detailed pre-clinical and clinical studies of the candidate are provided in Supplementary Table S4.

Comparative analyses on sera from exposed individuals before and after PZQ treatment known for altering schistosome-specific immune reactions qualitatively and quantitatively were performed to identify and characterize immunogenic Sh proteins (152). These studies show that GST is involved in detoxification and antioxidant pathways (144, 151). The protein is expressed on the tegument and sub-tegument of adult schistosomes of many species and plays a pivotal role in modulating the host’s immune response during infection. Inhibition of its enzymatic activities through neutralizing antibodies following immunization may be detrimental to the parasite. As key player of host-parasite interactions 28GST is an attractive vaccine candidate (143–151). It is a dimer of two very similar monomers, each having N- and C-terminal domains (144). Comparisons of Sh28GST and Sb28GST show that 75% of all residues are fully conserved among Sm, Sj, Sh, and *S. bovis* (Sb), suggesting a close evolutionary relationship (145, 151).

Initial research revealed that the passive transfer of 28GST-specific IgM antibodies with blocking effect of the enzymatic activity of Sm28GST in rodents resulted in worm decline, impairments in the female fecundity, and reduced egg viability related to laying, hatching and tissue deposition, suggesting that blocking effects of enzyme activities are detrimental to parasites (153). Interestingly, though PZQ has no pharmacological effect on the functional enzymatic activities of Sh28GST, its binding to Sj26GST results in a steric inhibition and altered immune responses and transport for large ligands. Whether upregulation or mutagenesis of parasitic GST could confer resistance to PZQ is debatable but may suggest the need for additional anti-schistosomal drugs (144, 151, 152, 154, 155).

Early pre-clinical experiments of Sh-infected *Papio anubis* baboons and *Erythrocebus patas* monkeys immunized with Sm28GST followed by homologous Sm or heterologous Sh challenge, and cattle vaccinated with Sm28GST with or without muramyl-di-peptide (MDP) or Sb28GST with or without MDP followed by Sh and *S. mattheei* challenge, showed heterologous immunity and protection post challenge with anti-fecundity and improved organ pathology. This suggests Sm28GST may have potential as polyvalent cross-protective vaccine candidate (145–148, 156, 157). In mice, Sh28GST elicited stronger IgA, IgG and IgE antibody titers compared to Sh-infected mice (158–160). Similar immune responses with predominant IgA, IgG and IgE antibodies were detected among infected humans from highly endemic Senegalese and Kenyan settings. IgE was positively correlated with older, lightly-infected subjects and negatively correlated with younger, highly-infected Zimbabweans (158–164). Naturally Sh-exposed Zimbabwean children from an endemic area demonstrated the highest and lowest Th2-cytokines among children aged 4–9 and 10–12 years of age, respectively, prior to PZQ treatment, and the highest Th1-/Th2-/Th17-cytokine levels among children aged 4–9 years six weeks post PZQ treatment (146).

Experiments using different expression vectors, e.g., *Bordetella pertussis*, *Mycobacterium bovis*, *Saccharomyces cerevisiae*, and *Salmonella Typhimurium*, and administration routes (intranasal, intraperitoneal and oral), among rodent and monkey models induced mixed Th1/Th2 responses with dose-dependent IgG1, IgG2a, IgG2b and IgA antibodies, and ADCC (148, 149, 164–167). Wild-caught *Erythrocebus patas* monkeys immunized with Sh28GST with CFA/IFA but not Sh28GST with Bacillus Calmette-Guérin adjuvant developed IgG and IgA titers with homogeneous anti-pathology processes, such as acute eosinic and chronic sclerotic inflammations, compared to heterogeneous processes in the alternate cohort (148). Dose-escalation toxicological assessments of Sh28GST adjuvanted with alum induced transient local inflammatory reactions and no clinical, anatomical or physiological modifications among rodents (168).

An initial Phase 1a trial assessed the safety, tolerability and immunogenicity of 100μg Sh28GST expressed in *Saccharomyces cerevisiae* and adjuvanted with alum followed by two booster doses on day 28 and 150 in healthy adult Caucasians 18–30 years of age (NCT01512277). A dose-escalation open-label trial of 300μg Sh28GST in alum with a single booster dose 4 weeks later was performed (168, 169). The vaccine was safe and well tolerated with only mild vaccine-related adverse events. Th2-type Sh28GST antibodies were induced by additional booster doses in a dose-independent manner. IgG1, IgG2 and IgG3 subclass antibodies, weak IgA and IgG4 titers, and no IgE antibodies, as well as a Th-1/Th-2 cytokine expression response, were detected. Inhibition of GST enzymatic activity was seen among all vaccine recipients and enhanced with additional doses coupled with the production of IgG1 and IgG3 subclass antibodies (143, 146, 168, 170). A subsequent Phase 1b trial in non-infected children 6–10 years of age (115) from schistosomiasis endemic Saint-Louis, Senegal, confirmed the vaccine safety and tolerability and the induction of high antibody titers (115, 143, 170). Bilharvax^®^ in combination with PZQ treatment was also safe in infected adults and children (171). These preliminary clinical findings led to the implementation of an efficacy trial.

A randomized, placebo-controlled Phase 3 trial investigated the safety, efficacy, long-term recurrences of clinical and parasitological manifestations, and immunogenicity of Bilharvax^®^ among Sh-infected school children 6–9 years of age in the Saint-Louis region of the Senegal River Basin, with high endemicity (>60%) (171, 172). Subsequent to clearance of ongoing schistosomiasis infection with two doses of PZQ prior and post immunization at week 44, children were randomized to receive three subcutaneous injections of either Bilharvax^®^ or Alhydrogel^®^ alone serving as control group at weeks 0, 4, and 8 followed by a booster dose at week 52, one year after the first injection (168, 171, 172). The primary endpoint was efficacy, evaluated as delay of recurrence of urinary schistosomiasis, and defined by a microhematuria associated with at least one living Sh egg from baseline to week 152. Bilharvax^®^ combined with PZQ was safe and well tolerated. No disease recurrence was observed among vaccinees during a median follow-up period of 76 and 92 weeks and 76 weeks among the vaccine and the control groups, respectively. However, at week 152, at least one recurrence of urinary schistosomiasis without differences in morbidity and parasitological manifestations assessed by ultrasonography was experienced by 86% and 85% of recipients in each group. Sh28GST-specific total IgG, IgG1, IgG2, IgG4 and modest IgE antibodies were elevated in the vaccine group, but unexpectedly IgG3 and IgA titers were absent in both groups. The immune response generated inhibited the enzymatic activity of Sh28GST preventing schistosomiasis pathology; ≥70% of sera in the vaccine group compared to 8% in controls had antibodies. The absence of specific IgG3 antibodies in the vaccine group, and the low IgA and IgE levels might represent a key factor involved in the lack of efficacy of the vaccine in this trial. Indeed, previous studies have shown elevated Sh28GST IgG3, IgE, and IgA antibodies compared to IgG1 in association with acquired immunity against reinfection to urinary schistosomiasis (173). Immuno-epidemiological studies in human populations indicate that the presence of IgG3 antibodies correlates with naturally acquired protective immunity against schistosomiasis (174). A Phase 1 clinical trial conducted in healthy subjects (168) revealed that Sh28GST induced IgG1 and high IgG3 titers, an effect that was associated previously with reduced egg production and decreased urinary tract pathology among Sh-infected individuals. Future trials should thus reassess Bilharvax^®^ in the absence of PZQ, and preferably in non-infected younger children utilizing another pro-Th1 adjuvant.

## METHODOLOGICAL CONSIDERATIONS

### Animal Models and Predictive Value of Protection in Humans

Among the experiments presented within the scope of this review, there are notable differences between the animal models, and parasitological and immunological assessments used - in particular for non-human primate challenge studies. These differences render comparisons between vaccine candidates and experimental outcomes difficult. For example, the Sh28GST candidate failed to confer efficacy in Phase 3 trials in humans while protection was demonstrated in the *Erythrocebus patas* monkey model, which in this case showed no positive predictive value. How would the *Papio anubis* baboons challenge model used for Sm-p80 compare to the *Erythrocebus patas* monkey model in terms of species susceptibility, cercarial challenge method, and immunogenicity assessment?

With the exception of a few antigens, the prophylactic efficacy of the majority of schistosome vaccine candidates has been evaluated only in the murine model which, inherently, appears to have an apparent ceiling of 40–50% protection (175). It is therefore advisable that while designing immunization regimens for clinical trials, data generated in the murine model should be used with caution. Baboons may serve as a useful bridge between mouse and human studies (8).

It is also noteworthy that two out of three vaccines candidates that progressed to advanced clinical development were not tested in non-human primate challenge models. The Sm14 vaccine entering into Phase 2 has not been tested in non-human primates so far. It is not clear whether Sm14 will undergo a challenge model experiment before entering an efficacy trial. Sm-TSP-2 vaccine was also not tested in non-human primates and it does not appear that this is being considered in preparation of a Phase 2b trial in Uganda.

### Parasitological Assessments

Across the evaluation of different schistosomiasis vaccines, there does not seem to be a general consensus about parameters to assess and the methods of measurement for non-human primate challenge experiments and efficacy trials in humans.

Parasitological post-challenge outcomes as primary efficacy endpoints are also difficult to interpret. In efficacy trials, it is assumed that egg output and hatching percentage would be a surrogate of worm burden to assess efficacy endpoints. The determination of worm burden is only possible in non-human primate models after portal perfusion. However, fecal egg output and/or circulating antigen levels are surrogate markers in humans. The sensitivity of these surrogates, the correlation between worm burden and egg output, and the validation of these surrogates in humans need further research.

In addition, the duration of protection is unknown since all baboons in the reported trials were sacrificed. In a challenge study in baboons vaccinated with Sm-p80, post challenge there are still ~7% of active female worms producing eggs with little pathogenicity (123). It remains unclear how this would change after longer periods of observation or repeated exposure.

### Immune Response Assessment and PZQ Impact

Across the evaluation of the different vaccine candidates, there does not seem to be consensus about the assessment of the following parameters: immune responses, including which variables to measure, and time points; in addition, consensus is needed on the method of measurement for non-human primate challenge experiments and efficacy trials in humans in terms of assay validation, standardization of reagents, and repository. As highlighted in the Krautz-Peterson’s study (62), using conformationally-native immunogens may be important when preparing antibodies for detection of schistosome tegumental antigens under natural conditions, such as for *in situ* localization or live worm staining.

PZQ may modulate transient and long-term immunological effects, which in turn may augment or antagonize vaccine-induced immune responses and skew towards a more pro-inflammatory response (47, 146, 176). In Zhang’s challenge study (123), the Sm-p80 vaccine exhibited potent prophylactic efficacy against transmission of Sm infection and was associated with significantly less egg-induced pathology compared to unvaccinated control animals. Specifically, the vaccine resulted in a 93.5% reduction female worms and impacted significantly the major clinical manifestations of hepatic and intestinal schistosomiasis by reducing the tissue egg-load by 90%. A 35-fold decrease in fecal egg excretion in vaccinated animals combined with an 81.5% reduction of egg hatching into the snail-infective stage (miracidia) demonstrates the parasite transmission-blocking potential of the vaccine. Higher Sm-p80 expression in female worms and Sm-p80-specific antibodies in vaccinated baboons appear to play an important role in vaccine-mediated protection. The immune correlates of protection are, however, not known (potential Th1 response with IFN-γ, IgG1 and IgG3)?. It must be noted that Ahmad’s work in non-human primates describes a mixed Th1-Th2 response without IgG3 antibodies. Little was learnt on immune correlates of protection however. Cell-mediated immune responses were not assessed.

Vaccination neither induces IgG4 nor a pro-inflammatory response after PZQ treatment and cercarial challenge. However, the reduction of worms and eggs after PZQ before challenge was not assessed (142). A clearer picture of these parasitological and immunological parameters might be generated from the comparison of the following groups: group 1 chronic infection, group 2 chronic infection receiving PZQ, group 3 chronic infection receiving PZQ with subsequent cercarial challenge, and group 4 chronic infection receiving PZQ followed by Sm-p80 vaccination and cercarial challenge.

To better understand field conditions of prior infection with schistosomes and post treatment with PZQ, baboons underwent Sm cercariae trickle infections over five weeks allowing the development of chronic disease and were treated subsequently with PZQ. After initial infection and during chronic disease, there was an increase in NK and NKT cells while the CD4:CD8 T-cell ratio inverted from 2:1 to 1:2.5. The cytokine expression in peripheral blood mononuclear cells after trickle infections was polarized more towards a Th2 response with a gradual increase in the Th1 profile in the chronic disease stage. Following PZQ treatment, immune cell populations reverted back towards naïve pre-treatment levels with the exception of an increase in B cells; however, expression of Th1, Th2, and Th17 cytokines was significantly increased. The implications of such findings for vaccine studies in baboons and humans remain unclear (177).

Natural history studies in areas targeted for efficacy trials seem essential, in particular in the context of PZQ MDA. Using well-established Brazilian cohorts of putative resistant and chronically-infected individuals stratified by the intensity of their Sm infection, arrays for IgG subclass and IgE responses were probed to these antigens to detect antibody signatures that were reflective of protective *vs*. non-protective immune responses. Moreover, probing for IgE responses allowed identifying antigens that might induce potentially deleterious hypersensitivity responses if used as subunit vaccines in endemic populations. Using multi-dimensional cluster analysis, the authors showed that putatively resistant individuals mounted a distinct and robust IgG1 response to a small set of newly discovered and well characterized surface tegument antigens compared to chronically infected individuals who mounted strong IgE and IgG4 responses to many antigens (178).

In another set of studies, Sm-infected individuals recruited from a schistosomiasis endemic area in Uganda, were treated with PZQ and followed up from five weeks to one-year post-treatment. Pre-treatment and five weeks post-treatment IgE, IgG1 and IgG4 levels against recombinant schistosomula antigens SmKK7, SmLy6A, SmLy6B and SmTSP7 were measured. Being male was associated with higher pre-treatment IgG1 levels to SmKK7, SmLy6a and Sm adult worms. There was no consistent association between the detectable five weeks post-treatment antibody responses against schistosomula antigens and reinfection intensity one year after PZQ treatment. Sm-infected individuals exhibited detectable antibody responses to schistosomula antigens that were affected by treatment. These findings indicate that schistosomula antigens induce highly heterogeneous antibody responses following treatment besides prior exposure as well as poly-parasitism and other co-infections. Some antigens induce an immune response and give rise to increased antibodies, while other antigens do not, and this could have implications for vaccine development (47, 179). More natural infection studies are needed to elucidate the immune responses before and after PZQ administration in geographical areas where vaccine efficacy trials are planned. Finally, although the latest non-human primate studies published (123, 142) use sophisticated immune assessments, the statistical analysis of baboon immunological data may benefit from new tools such as COMPASS, a computational framework for unbiased combinatorial polyfunctionality analysis of antigen-specific T-cell subsets, used in HIV vaccine trials (180).

### Controlled Human Infection Model

Controlled human infection models (CHIM) are gaining recognition as an approach to accelerating vaccine development, for use in both non-endemic and endemic populations. CHIM could possibly guide the identification of a promising candidate vaccines for further trials and advance the understanding of protective immunity. The Sm CHIM model developed in the Netherlands (181) might be an important vaccine assessment tool providing guidance without being a gatekeeper of clinical efficacy trials. Because responses to infections and candidate vaccines are likely to differ between endemic and non-endemic settings, it has been proposed to establish a Sm-CHIM in Uganda where also Sh is endemic (182). A first dose-escalating clinical safety trial in 17 volunteers using male Sm cercariae, which do not produce eggs and therefore do not cause lasting pathology, as a challenge was recently conducted in Leiden, the Netherlands (NCT02755324). The primary endpoints were adverse events and infectivity. A dose-related increase in adverse events due to acute schistosomiasis syndrome, occurred in nine of 17 volunteers. Overall, five volunteers reported severe adverse events (SAEs). Infection with 20 Sm cercariae led to SAEs in 18% of volunteers and high infection rates. Worm-derived circulating anodic antigen, the biomarker of the primary infection endpoint, peaked in 82% of volunteers during 3 to 10 weeks post exposure. All volunteers showed IgM and IgG1 seroconversion and worm-specific cytokine production by CD4+ T cells. All volunteers were cured with PZQ provided at 12 weeks after exposure (183).

This model is not validated and its positive and negative predictive values of the efficacy outcome of vaccines in humans remain unclear. Also, it does not reflect the situation of endemic areas in terms of age of target population, repeated natural exposure, and PZQ rollout. It remains unclear what parasitological and immunological parameters would be assessed with the exception of serum circulating anodic antigen.

## CONCLUSIONS

Schistosomiasis remains a neglected tropical disease of major public health concern with high levels of morbidity globally. Despite considerable efforts in MDA programs, schistosomiasis is still not contained. A schistosomiasis vaccine may be a critical component of a multifaceted prevention control approach. Four major vaccine candidates have entered advanced pre-clinical and clinical development, but only one reached Phase 3 in Africa and failed to confer meaningful efficacy. Several other promising candidates are in preclinical and early clinical development. This assessment of current candidates revealed some methodological issues that preempt a predictive comparison between leads. These include variability in animal models, in particular, non-human primate studies, and their predictive value of protection in humans, lack of consensus on essential parasitological and immunological assessment parameters and of reliable surrogate markers of protection, and the need for better designed parasitological and immunological natural history studies in the context of PZQ. Although the CHIM model is a great initiative, it needs to be validated for its positive and negative predictive values for vaccine efficacy in endemic settings and should not be considered as a gatekeeper of field trials in humans.

More research is also needed to test new potential vaccine antigens such as cholinesterases regulating the neurotransmission of *Schistosoma* (184), or new tegumental proteins identified by microarray and other modern technologies (178, 185–189), and more potent adjuvants targeting specific parts of the innate immune system to tailor a protective immune response for lead schistosome vaccine candidates with the long-term aim to achieve high levels of worm reduction and ultimately elimination of this terrible disease.

## Supplementary Material

Table 4

Table 2

Table 3

Table 1
