# Supplementary material for: Recent Advances and Methodological Considerations on Vaccine Candidates for Human Schistosomiasis
Source: Front Trop Dis. Author manuscript; Available in PMC 2024 Sep 13. (PMC11392908; doi:10.3389/fitd.2021.719369)
Supplement: Table 4 [file NIHMS2017111-supplement-Table_4.docx]

**S4 Table.** Pre-clinical and clinical development of Sh28GST

| **Formulation** | **Design** | **Endpoints** | **Reference** |
| --- | --- | --- | --- |
| **Antigen:** rSh28GST (expression system: *S. cerevisiae)*  **Adjuvant:** Alhydrogel | **Experimental model:** humans (male and female children 6-9 years *S. haematobium* infected from highly endemic area of Saint-Louis region, Senegal River valley, Senegal)  **Administration:** immunization: s.c.  *Phase III 2-arm safety, efficacy, pathology and immunogenicity study among infected school children (pre-treatment with 2 doses PZQ prior to vaccination and third booster)*  **Immunization VG:** 100ug rSh28GST/Alhydrogel followed by 3 boosters of 100ug rSh28GST/Alhydrogel at week 4, 8 and 52 (CG: 0.4ml Alhydrogel); follow-up for up to 152 weeks | **Participation:** as per study protocol  **Safety:** 9 SAEs not treatment-related and not life threatening; 47% AEs grade 1, 6% AEs of grade ≥3; 74% in VG, 58% in CG  **Pathogenicity:** median follow-up without recurrence 22.9 months in VG and 18.8 months in CG; ≥1 recurrence 86.4% in VG and 89.6% in CG at week 152; recurrence 84.8% in VG and 89.6% in CG during week 65-152  **Immunogenicity:** total IgG, IgG1, IgG2, IgG4 and weak IgE in VG; ≥70% neutralizing antibodies in VG impacting on enzymatic activity | Riveau G, et al., 2018,  NCT00870649 [173,174] |
| **Antigen:**  rSh28GST (expression system: *S. cerevisiae*)  **Adjuvant:** Alhydrogel | **Experimental model:** Humans (healthy Caucasian male adults 18-30 years)  **Administration:** immunization: s.c.  *Phase I safety, tolerability and immunogenicity study among healthy male adults*  **Immunization VG1** (double-blind)**:** 100ug rSh28GST+0.5ml Alhydrogel followed by 2 boosters of 100ug rSh28GST+0.5ml Alhydrogel at day 28 and 150 (CG: 0.5ml alum)  *Phase I dose-escalation safety, tolerability, immunogenicity study among healthy male adults*  **Immunization VG2** (open-label)**:** 300ug rSh28GST+ Alhydrogel followed by 1 booster of 300ug rSh28GST+ Alhydrogel at day 28 | **Participation:** no drop-out  **Safety:** no vaccine-related SAEs; minor clinical and biological vaccination-related AEs  **Immunogenicity:** strong IgG1, IgG2 and IgG3, but weak IgG4 and IgA and no IgE in VGs1-2; mononuclear cells proliferated for IL-2, Il-5, IL-10, IL-12, IL-13, IFN-𝛾 and TGF-𝛽 in VG1; dose-dependent inhibition of enzymatic activity in VGs1-2 correlated with IgG1 and IgG3 | Riveau G, et al., 2012,  NCT01512277 [170,171] |
| **Antigen:**  pTECH2-Sh28GST/TetC, pTECH10-Sh28GST (expression system: *E. coli* TG2, *S. Typhimurium* SL5338, SL3261,)  **Adjuvant:** TetC | **Experimental model:** BALB/c mice  **Administration:** oral  **Immunization:** 4x10^10^-5x10^10^ CFU of pTECH2-Sh28GST/TetC or pTECH10-Sh28GST in PBS | **Immunogenicity:** IgG1, IgG2A, IgG2B and IgA but no IgG3 and IgM with IFN-𝛾, IL-2 and IL-4 starting from week 3 particular with TetC; neutralizing antibodies with TetC adjuvant inhibited 85% of GST’s enzymatic activity | Lee JJ, et al., 2000  [151] |
| **Antigen:**  rSh28GST (experimental system: *S. cerevisiae* TGY73.4)  **Adjuvant:** BCG, CFA/IFA | **Experimental model:** patas monkey (*Erythrocebus patas*, wild-caught)  **Administration:** immunization: i.d. (BCG), s.c. (CFA); challenge p.c.  **Immunization:** 100ug rSh28GST+0.1ml BCG or CFA followed by 1 booster dose of 100ug rSh28GST+0.1ml BCG or IFA on day 42 (CG: BSA+BCG or CFA/IFA); challenge with 1500 *S. haematobium* cercariae (Libore strain, Niger) on day 154; death | **Worm reduction:** females by 1/3 with CFA  **Egg reduction:** significant for urinary, fecal and tissue eggs in both groups; homogeneous tissue inflammation with CFA (63% improvement); heterologous tissue inflammation with BCG (66% improvement)  **Immunogenicity:** total IgG and IgA with CFA; weak total IgG and IgA with BCG | Boulanger D, et al., 1999 [150] |
| **Antigen:**  rBCG-pENSh28GST, rBCGpEN005, rBCG (expression system: *M. bovis* 1173P2) | **Experimental model:** BALB/c mice  **Administration:** immunization: i.p., i.n.  **Immunization VG1** (i.p.)**:** 10^8^, 5x10^6^, or 5x10^5^ rBCG or rBCG-pENSh28GST in PBS followed by 1 booster dose of 10^8^, 5x10^6^, or 5x10^5^ rBCG or rBCG-pENSh28GST on day 56  **Immunization VG2** (i.n.)**:** 10^7^ rBCG or rBCG-pENSh28GST or rBCGpEN005 followed by 1 booster dose of 10^7^ rBCG or rBCG-pENSh28GST on day 112 | **Immunogenicity** (i.p.)**:** strongest anti-rBCG in 10^8^, lower anti-rBCG in 5x10^6^ and no anti-rBCG 5x10^5^ with 1^st^ dose; strong increase of anti-rBCG in 5x10^6^ and 5x10^5^ but weak increase of anti-rBCG in 10^8^ with 2^nd^ dose; strong increasing anti-rBCG-pENSh28GST with increasing doses (booster)  **Immunogenicity** (i.n.)**:** strong increasing anti-rBCG and anti-rBCG-pENSh28GST (i.e. IgG1, IgG2A, IgG2B and IgA in sera and lung lavage) with increasing doses (booster); no anti-Sh28GST without rBCG; low titer of anti-rBCGpEN005 versus high titer of anti-rBCG-pENSh28GST though Sm28GST and Sh28GST share 90% amino acid identity; dose-dependent inhibition of enzymatic activity due to anti-rBCG-pENSh28GST; no altered delayed type hypersensitivity due to anti-rBCG-pENSh28GST | Kremer L, et al., 1998  [167] |
| **Antigen:**  rSh28GST  **Adjuvant:** CFA | **Experimental model:** CBA/Ca mice  **Administration:** infection: p.c.; immunization: s.c.  **Infection 1:** 90 *S. haematobium* cercariae followed by 2 boosters of 90 *S. haematobium* cercariae each on day 7 and 14; sampling each on week 6, 10, 14, 18, 22 and 27  **Infection 2:** 300 *S. haematobium* cercariae; sampling each on week 6, 8, 10, 14 and 16  **Immunization:** 50ug rSm28GST+ 80ul CFA followed by 1 booster of 50ug rSm28GST+ 80ul CFA on day 14 | **Immunogenicity:** splenocytes and mesenteric lymph node cells proliferated for IFN-𝛾, IL-4 and IL-10 for SEA and SWA (but decline up to week 27) but not for rSh28GST; IgE and IgA and total IgG increase with egg production similarly for SEA, SWA and rSh28GST | Lane A, et al., 1998  [162] |
| **Antigen:** rSm28GST (expression system: *E. coli* TGE901)  **Adjuvant:** MDP | **Experimental model:** wild patas monkeys (*Erythrocebus patas*)  **Administration:** immunization: s.c.; challenge: p.c.  **Immunization:** 100ug rSm28GST followed by 1 booster of 100ug rSm28GST on day 14 (CG: MDP); challenge with 1,500 *S. haematobium* cercariae (Libore strain, Niger) on day 49; death | **Egg reduction:** 62-83% urine; 86-93% feces; ~60% miracidia hatching; intra-uterine >30%; few polypoid lesions, hemorrhagic foci and moderate fibrosis in urinary balder wall  **Immunogenicity:** IgA and total IgG (increasing following booster; decreasing from week 23 post infection) | Boulanger D, et al., 1995 [149] |

Abbreviations: GST=glutathione s-transferase; VG=vaccine group; CG=control group; i.d.=intradermal; s.c.=subcutaneous; i.n.=intranasal; i.p.=intraperitoneal; p.c.=percutaneous; *S. haematobium*/Sh=Schistosoma haematobium; SAE=serious adverse event; AE=adverse event; GST=glutathione s-transferase; MDP=muramyl-di-peptide; SEA=soluble egg antigen; SWAP=soluble adult worm protein; CFA=complete Freund’s adjuvant; IFA=incomplete Freund’s adjuvant; *M. bovis*=*Mycobacterium bovis*; PBS=phosphate buffered saline; BCG=Bacillus Calmette Guérin; BSA=bovine serum albumin; *E. coli*=*Escherichia coli*; *S. cerevisiae*=*Saccharomyces cerevisiae*; *Salmonella Typhimurium*=*S. Typhimurium*; CFU=colony forming unit; alum=aluminum hydroxide; TetC=atoxic fragment C of tetanus toxin; Alhydrogel=aluminum hydroxide salt; Ig=immunoglobulin; IFN-𝛾=interferon gamma; IL=interleukin; TGF-𝛽=transforming growth factor beta; PZQ=praziquantel.

Note: References were obtained through systematic searches in PubMed without restrictions in language and time, including a reference search among the publications included in this review, and at the U.S. National Library of Medicine for clinical trial; the last searches were performed on April 25, 2021.
