# Supplementary material for: Recent Advances and Methodological Considerations on Vaccine Candidates for Human Schistosomiasis
Source: Front Trop Dis. Author manuscript; Available in PMC 2024 Sep 13. (PMC11392908; doi:10.3389/fitd.2021.719369)
Supplement: Table 2 [file NIHMS2017111-supplement-Table_2.docx]

**S2 Table.** Pre-clinical and clinical development of Sm14

| **Formulation** | **Design** | **Endpoints** | **Reference** |
| --- | --- | --- | --- |
| **Antigen:** rSm14  **Adjuvant:** GLA-SE | **Experimental model:** humans (healthy and *S. mansoni* and/or *S. haematobium* infected schoolchildren 8-11 years from endemic Saint-Louis region, Senegal River valley, Senegal)  **Administration:** immunization: i.m.  *Phase IIb 3-arm safety and immunogenicity study among healthy and infected school children (pre-treatment with 1 dose PZQ 2-4 weeks prior to vaccination)*  **Immunization VG1:** 50ug rSm14+2.5ug GLA-SE followed by 2 boosters of 50ug rSm14+2.5ug GLA-SE each on day 28 and 56; follow-up for up to 90 days 🡪 *healthy children*  **Immunization VG2:** 50ug rSm14+2.5ug GLA-SE followed by 2 boosters of 50ug rSm14+2.5ug GLA-SE each on day 28 and 56; follow-up for up to 90 days 🡪 *S. mansoni and/or S. haematobium infected children (pre-treatment with 1 dose PZQ 2-4 weeks prior to vaccination)*  **Immunization VG3:** no vaccine 🡪 *S. mansoni and/or S. haematobium infected children (pre-treatment with 1 dose PZQ)* | Completed; not yet published | NCT03799510 [117] |
| **Antigen:** rSm14  **Adjuvant:** GLA-SE | **Experimental model:** humans (healthy male adults 18-45 years from highly *S. mansoni* and *S. haematobium* endemic area of Saint-Louis region, Senegal River valley, Senegal)  **Administration:** immunization: i.m.  *Phase IIa dose-escalation safety and immunogenicity study among healthy exposed adults (pre-treatment with 1 dose PZQ 3 weeks prior to vaccination)*  **Immunization VG1:** 50ug rSm14+2.5ug GLA-SE followed by 2 boosters of 50ug rSm14+2.5ug GLA-SE each on day 28 and 56; follow-up for up to 90 days  **Immunization VG1:** 50ug rSm14+5.0ug GLA-SE followed by 2 boosters of 50ug rSm14+5.0ug GLA-SE each on day 28 and 56; follow-up for up to 90 days | Completed; not yet published | NCT03041766 [116] |
| **Antigen:** rSm14 (expression system: *P. pastoris*)  **Adjuvant:** GLA-SE | **Experimental model:** humans (healthy male and non-pregnant, non-breastfeeding female adults 18-49 years from non-endemic area Brazil)  **Administration:** immunization: i.m.  *Phase I trial on safety and immunogenicity of rSm14/with/GLA-SE among healthy non-exposed adults*  **Immunization:** 50ug rSm14+10ug GLA-SE followed by 2 boosters of 50ug rSm14+10ug GLA-SE each on day 30 and 60; follow-up for up to 120 days | **Participation:** 100% 1^st^ dose, 90% 2^nd^ dose, 85% 3^rd^ dose; 2 subjects dropped out  **Safety:** no SAEs; no non-related clinical and laboratory events; AEs e.g. local pain (85% 1^st^ dose, 50% 2^nd^ dose, 35% 3^rd^ dose), erythematous reactions (11%), tenderness (5%) and slight temperature (5%)  **Immunogenicity:** total IgG among all subjects that increased in 88% of subjects following 2^nd^ dose; steady increase of total IgG, IgG1, IgG2, IgG3 an IgG4 starting from day 30; no IgE; PBMCs proliferated for IL-2, IL-5, IL-10, IFN-𝛾 and TNF-𝛼 | Santini-Oliveira M, et al., 2016; NCT01154049 [112,113] |
| **Antigen:** pCI/Sm14, pVAX1/Hsp65 (expression system: *E. coli* DH5a) | **Experimental model:** C57BL6/6 mice  **Administration:** immunization: i.m.; challenge: s.c.  **Immunization VG1:** 100ug pCI/Sm14 followed by 3 boosters of 100ug pCI/Sm14 each on day 7, 14 and 21; challenge with 20 *S. mansoni* cercariae (LE strain) on day 36; death on day 51, 84 and 105  **Immunization VG2:** 100ug pVAX1/Hsp65 followed by 3 boosters of 100ug pVAX1/Hsp65 each on day 7, 14 and 21; challenge with 20 *S. mansoni* cercariae (LE strain) on day 36; death on day 51, 84 and 105  **Immunization VG3:** 2ug pCI/Sm14+pVAX1/Hsp65 followed by 3 boosters of 2ug pCI/Sm14+pVAX1/Hsp65 each on day 7, 14 and 21; challenge with 20 *S. mansoni* cercariae (LE strain) on day 36; death on day 51, 84 and 105 | **Worm reduction:** 28-35% in VG1; 0% in VG3  **Egg reduction:** 97% and 60% dead intestinal tissue eggs in VG3 and VG1, respectively; reduction of collagen and hepatic stellate cells around granuloma in VG3  **Immunogenicity:** highest ratio of memory CD8+ T-lymphocytes to number of CD8+ cells in splenocytes of VG3; enhanced IgG1/IgG2A ratio in VG3 | Espíndola MS, et al., 2014 [111] |
| **Antigen:** pQE31-Sm14, pQE31-TrSm29 (expression system: *E. coli* M15 (pREP4)  **Adjuvant:** poly(I:C) | **Experimental model:** Swiss albino mice  **Administration:** immunization: i.p.; challenge: p.c.  **Immunization VG1:** 20ug pQE31-Sm14±50ug poly(I:C) followed by 2 boosters of 20ug pQE31-Sm14±50ug poly(I:C) each on day 14 and 28 (CG: PBS, poly(I:C)); challenge with 100 *S. mansoni* cercariae on day 42; death on day 91  **Immunization VG2:** 10ug pQE31-TrSm29±50ug poly(I:C) followed by 2 boosters of 10ug pQE31-TrSm29±50ug poly(I:C) each on day 14 and 28 (CG: PBS, poly(I:C)); challenge with 100 *S. mansoni* cercariae on day 42; death on day 91  **Immunization VG3:** 20ug pQE31-Sm14/10ug pQE31-TrSm29±50ug poly(I:C) followed by 2 boosters of 20ug pQE31-Sm14/10ug pQE31-TrSm29±50ug poly(I:C) each on day 14 and 28 (CG: PBS, poly(I:C)); challenge with 100 *S. mansoni* cercariae on day 42; death on day 91 | **Worm reduction:** 69% and 68% ±poly(I:C) in VG3; 40% ±poly(I:C) in VG2 (liver)  **Egg reduction:** 31% and 40% ±poly(I:C) in VG3 (liver); 50% and 58% ±poly(I:C) in VG3 (intestine); 29% ±poly(I:C) in VG1; 32% ±poly(I:C) in VG2; fewer and smaller granuloma ±poly(I:C) in VG3  **Immunogenicity:** IgG1 but no IgG2A ±poly(I:C) in VG1-3 | Ewaisha RE, et al., 2015, 2014; Mossallam SF, et al., 2015 [104-106] |
| **Antigen:** Sm14(32-48), Sm14(53-69), Para(6-22), Para(210-226), Para(355-371)  **Adjuvant:** CFA/IFA | **Experimental model:** C57BL/6 mice  **Administration:** immunization: s.c.; challenge: p.c.  **Immunization:** Sm14 or Para or 10ug each Sm14(32-48)+Sm14(53-69)+Para(6-22)+Para(210-226)+Para(355-371)+10ug PADRE+100ul PBS+100ul CFA followed by 2 boosters of Sm14 or Para or 10ug each Sm14(32-48)+Sm14(53-69)+Para(6-22)+Para(210-226)+Para(355-371)+10ug PADRE+100ul PBS+100ul IFA each on day 15 and 30 (CG: 10ug PADRE+100ul PBS+100ul CFA/IFA or PBS or 25ug Sm14); challenge with 30 *S. mansoni* cercariae (LE strain) on day 45; death on day 90 | **Worm reduction:** 28-29% for peptide mix; 26-37% for Sm14; no significant reduction for Para  **Egg reduction:** 46% for peptide mix; 67% for Sm14; 43% liver granuloma area and 52% number of granuloma for peptide mix; 54% liver granuloma area and 61% number of granuloma for Sm14; no significant reduction in granuloma area and number for Para  **Immunogenicity:** total IgG for all peptides; lower IgG1/GG2 ratio Sm14 than Para and peptide mix; splenocytes highly proliferated for IFN-𝛾 in Sm14 (Th-1) and IL-4 and IL-10 in Para (Th-2) | Garcia TC, et al., 2008  [103] |
| **Antigen:** pCI/rSm14 (expression system: *E. coli* DH5a)  **Adjuvant:** IL-12 | **Experimental model:** C57BL/6 mice  **Administration:** immunization: i.m.; challenge: p.c.  **Immunization:** 100ug pCI or 100ug pCl/rSm14 or 50ug pCI+50ugpCI/IL12 or 50ug pCI/rSm14+50ug pCI/IL12 followed by 3 boosters of 100ug pCI or 100ug pCl/rSm14 or 50ug pCI+50ugpCI/IL12 or 50ug pCI/rSm14+50ug pCI/IL12 each on day 15, 30 and 45; challenge with 30 *S. mansoni* cercariae (LE strain) on day 60; death on day 105 | **Worm reduction:** 41% in pCl/rSm14, but no enhancement in pCI/rSm14+pCI/IL12  **Egg reduction:** no difference in granuloma formation and tissue fibrosis in pCl/rSm14 and pCI/rSm14+pCI/IL12  **Immunogenicity:** total IgG titer in pCl/rSm14 but not in pCI/rSm14+pCI/IL12; splenocytes proliferated for IFN-𝛾, IL10, low TNF-𝛼 and no IL-4 in pCl/rSm14 and pCI/rSm14+pCI/IL12; CD8+T-cells/BAL producing IFN-𝛾 lower in pCI/rSm14+pCI/IL12 | Fonseca CT, et al., 2006 [101] |
| **Antigen:** pMAL-rSm14 (expression system: *E. coli* DH5a, *S. Typhimurium* SL3261)  **Adjuvant:** IL-12, UFV-H2b20 (expression system: *L. delbrueckii* UFV-H2b20, *L. lactis* NZ9000) | **Experimental model:** Swiss mice  **Administration:** immunization: p.c.; challenge: t.c.  **Immunization** (BALB/c)**:** 100ul 10^9^ *L. delbrueckii*-PBS (UFV-H2b20) or 100ul 10^9^ *S. Typhimurium*-pMAL-rSm14-PBS or 100ul 10^9^ *L. lactis*-PBS (IL-12) or 100ul 10^9^ *S. Typhimurium*-pMAL-rSm14-PBS+*L. delbrueckii*-PBS (UFV-H2b20) or 100ul 10^9^ *S. Typhimurium*-pMAL-rSm14-PBS+*L. lactis*-PBS (IL-12) followed by boosters of 100ul 10^9^ *L. delbrueckii*-PBS (UFV-H2b20) or 100ul 10^9^ *S. Typhimurium*-pMAL-rSm14-PBS or 100ul 10^9^ *L. lactis*-PBS (IL-12) or 100ul 10^9^ *S. Typhimurium*-pMAL-rSm14-PBS+*L. delbrueckii-PB*S (UFV-H2b20) or 100ul 10^9^ *S. Typhimurium*-pMAL-rSm14-PBS+*L. lactis*-PBS (IL-12) each on day 1, 2, 14, 15, 28 and 29; challenge with 50 *S. mansoni* cercariae (LE strain) on day 45; death on day 87 | **Worm reduction:** 35-49% disregarding of adjuvant  **Egg reduction:** 44% in *S. Typhimurium*-pMAL-Sm14-PBS; 45% in *S. Typhimurium*-pMAL-Sm14-PBS+*L. delbrueckii*-PBS (UFV-H2b20); 52% in *S. Typhimurium*-pMAL-Sm14-PBS+*L. lactis*-PBS (IL-12) (intestine); general reduction in hepatic granuloma size  **Immunogenicity:** total IgG, but no IgA among *S. Typhimurium*-pMAL-Sm14-PBS disregarding of adjuvant | Pacheco LG, et al., 2005, 2008  [109,110] |
| **Antigen:** pAE-TTFC/Sm14, TTFC, SM14 (expression system: *E. coli* DH5a, *E. coli* BL21-SI)  **Adjuvant:** alum | **Experimental model:** BALB/c mice, Swiss mice  **Administration:** immunization: s.c./p.c.; challenge: s.c.  **Immunization** (BALB/c)**:** 10ug TTFC+alum or 10ug Sm14+alum or 10ug pAE-TTFC/Sm14+alum or 10ug TTFC+Sm14+alum followed by 1 booster of 10ug TTFC or 10ug TTFC+alum or 10ug Sm14+alum or 10ug pAE-TTFC/Sm14+alum or 10ug TTFC+Sm14+alum on day 14 (CG: PBS+alum, TT+alum, Sm14); challenge with 10-times MLD of TTFC (protected if surviving after 96hrs) day 28; death on day 90  **Immunization** (Swiss)**:** 10ug Sm14+alum or 10ug pAE-TTFC/Sm14+alum or 10ug TTFC+Sm14+alum or 10ug TTFC+alum followed by 2 boosters of 10ug Sm14+alum or 10ug pAE-TTFC/Sm14+alum or 10ug TTFC+Sm14+alum or 10ug TTFC+alum on day 7 and 14 (CG: PBS+alum); challenge with 100 *S. mansoni* cercariae (BH strain) on day 74; death on day 119 | **Immunogenicity** (BALB/c): high anti-TT IgG and IgG1, but weak IgG2A among TTFC, pAE-TTFC/Sm14 and TTFC+Sm14; survival following MLD challenge without signs of disease in TTFC, pAE-TTFC/Sm14, TTFC+Sm14 and TT  **Immunogenicity** (Swiss): high anti-TT IgG, IgG1 and IgG2B, but weak IgG2A among TTFC, pAE-TTFC/Sm14 and TTFC+Sm14  **Worm reduction** (Swiss): 51% in Sm14; 51% in pAE-TTFC/Sm14; 35% in TTFC+Sm14 | Abreu PA, et al., 2004  [108] |
| **Antigen:** rBCG-pPL73-Sm14, BCG (expression system: *E.coli* DH5a), BCG 1172P2)  **Adjuvant:** alum | **Experimental model:** BALB/c mice  **Administration:** immunization: p.c.; challenge: s.c.  **Immunization VG:** 10^6^CFU/0.5 ml BCG or rBCG-pPL73-Sm14 (CG: saline, 10ug rSm14+alum) followed by 2 boosters on day 35 and 49; challenge with 100 *S. mansoni* cercariae on day 109; death on day 154 | **Immunogenicity:** no anti-rSm14 (confirmed with sub-immunizing dose); splenocytes proliferated for weak non-specific IFN-𝛾 and IL-4 (immunization of outbred Swiss mice with rBCG-pPL73-Sm14 revealed 48% worm reduction; no enhancement with additional boosters) | Varaldo PB, et al., 2004, 2006 [102,107] |
| **Antigen:** rSm14 (expression system: *E.coli* DH5a)  **Adjuvant:** CFA/IFA, alum, IL-12, alum+IL-12 | **Experimental model:** C57BL/6 mice  **Administration:** immunization: s.c.; challenge: p.c.  **Immunization VG1:** 25ug rSm14+CFA followed by 2 boosters of 25ug rSm14+IFA each on day 15 and 30 (CG: MBP/PBS, rSm14); challenge with 30 *S. mansoni* cercariae (LE strain) on day 45; death on day 90  **Immunization VG2:** 25ug rSm14+alum followed by 2 boosters of 25ug rSm14+alum each on day 15 and 30 (CG: MBP/PBS, rSm14); challenge with 30 *S. mansoni* cercariae (LE strain) on day 45; death on day 90  **Immunization VG3:** 25ug rSm14+IL-12 followed by 2 boosters of 25ug rSm14+IL-12 each on day 15 and 30 (CG: MBP/PBS, rSm14); challenge with 30 *S. mansoni* cercariae (LE strain) on day 45; death on day 90  **Immunization VG4:** 25ug rSm14+alum+IL-12 followed by 2 boosters of 25ug rSm14+alum+IL-12 each on day 15 and 30 (CG: MBP or PBS); challenge with 30 *S. mansoni* cercariae (LE strain) on day 45; death on day 90 | **Worm reduction:** 25% in VG1; 42% in VG3; 0% in VG2 and VG4  **Egg reduction:** significant decrease in granuloma in VG3  **Immunogenicity:** total IgG in VGs1-4; high IgG1 and low IgG2 in VG2; low IgG1 and high IgG2 in VG4; high IgG2 in VG1 and VG3; splenocytes proliferated for IFN-𝛾, IL-10 and TNF-𝛼 in VG3 | Fonseca CT, et al., 2004 [99] |
| **Antigen:** pRSET-His-rSm14 (expression system: *E.coli*) | **Experimental model:** Swiss mice  **Administration:** immunization: p.c.; challenge: s.c.  **Immunization VG1:** 10ug pRSET-His-rSm14 followed by 2 boosters of 10ug pRSET-His-rSm14 each on day 7 and 21 (CG: PBS); challenge with 100, 500 or 1000 *S. mansoni* cercariae (LE strain) on day 76; death of 50% of mice on day 130 while 50% remain under observation  **Immunization VG2:** 20ug pRSET-His-rSm14 followed by 2 boosters of 20ug or pRSET-His-rSm14 each on day 7 and 21 (CG: PBS); challenge with 100 *S. mansoni* cercariae (LE strain) on day 76; death of 50% of mice on day 130 while 50% remain under observation | **Worm reduction:** 37% and 50% with 100 cercariae in VGs1-2; 0% and 41% with 500 or 1,000 cercariae in VGs1-2  **Mortality rate:** 14% with 100 cercariae in VG2; 43% with 500 cercariae in VG2; 82% with 1,000 cercariae in VG2  **Immunogenicity:** no significant IgA and IgG among VGs1-2, but significant IgM in VG2 challenged with 500 or 1,000 cercariae | Ribeiro F, et al., 2002  [85] |
| **Antigen:** pRSETA-Sm14, pRSET-His-Sm14  (expression system: *E.coli* BL21 (DE3))  **Adjuvant:** MPL-TDM Ribi | **Experimental model:** Swiss mice  **Administration:** immunization: p.c.; challenge: s.c.  **Immunization VG1:** 10ug pRSETA-Sm14 or 10ug pRSET-His-Sm14 ± MLP-TDM Ribi followed by 2 boosters of 10ug pRSETA-Sm14 or 10ug pRSET-His-Sm14 ± MLP-TDM Ribi each on day 7 and 14 (CG: SE-PBS); challenge with 100 *S. mansoni* cercariae on day 74; death on day 119  **Immunization VG2:** 30ug pRSETA-Sm14 or 30ug pRSET-His-Sm14 ± MLP-TDM Ribi followed by 2 boosters of 30ug pRSETA-Sm14 or 30ug pRSET-His-Sm14 ± MLP-TDM Ribi each on day 7 and 14 (CG: SE-PBS); challenge with 100 *S. mansoni* cercariae on day 74; death on day 119 | **Worm reduction:** 40-55%  **Immunogenicity:** pRSETA-Sm14 and pRSET-His-Sm14 equally effective in protection with/without MPL-TDM Ribi | Ramos CR, et al., 2001  [94] |
| **Antigen:** rSm14-pGEMEX-1 (expression system: *E.coli* BL21 (DE3))  **Adjuvant:** CFA | **Experimental model:** New Zealand white outbred rabbits, outbred Swiss mice  **Administration:** immunization: s.c.; challenge: p.c., oral  **Immunization VG1** (rabbits)**:** 80ug rSm14+CFA followed by 2 boosters of 80ug rSm14+CFA on day 7 and of 80ug rSm14 on day 21 (CG: 600ug SE); challenge with 1,000 *S. mansoni* cercariae on day 81; death on day 126  **Immunization VG2** (mice)**:** 10ug rSm14+CFA followed by 2 boosters of 10ug rSm14+CFA on day 7 and 10ug rSm14 on day 21 (CG: 300ug SE); challenge with 100 *S. mansoni* cercariae on day 81 and death on day 126 or challenge with 3 *F. hepatica* metacercariae on day 66 and death on day 96 | **Worm reduction:** ≤66% in VG1 with ≤10 worms; ≤89% in VG2 with ≤10 worms; 100% protection against *F. hepatica* metacercariae in VG2  **Egg reduction:** reduced periportal infiltrate, mononuclear cells and periovular granulomas/necrosis in VG2 challenged with *S. mansoni* cercariae; no intraparenchymal hepatic but capsular cicatricial lesions in VG2 challenged with *F. hepatica* metacercariae  **Immunogenicity:** high level of protection among mice and rabbits vaccinated with/without CFA | Tendler M, et al., 1996  [87] |

Abbreviations: VG=vaccine group; CG=control group; p.c.=percutaneous; i.m.=intramuscular; s.c.=subcutaneous; t.c.=transcutaneous; i.p.=intraperitoneal; CFA=complete Freund’s adjuvant; IFA=incomplete Freund’s adjuvant; poly(I:C)=polyinosinic-polycytidylic acid sodium salt; alum=aluminum hydroxide; *F. hepatica*=*Fasciola hepatica*; *S. mansoni*/Sm=*Schistosoma mansoni*; *S. haematobium*=*Schistosoma haematobium*; SE=saline extract**;** *P. pastoris*=*Pichia pastoris*; *E. coli*=*Escherichia coli*; *S. Typhimurium*=*Salmonella Typhimurium*; *L. delbrueckii*=*Lactobacillus delbrueckii*; *L. lactis*=*Lactobacillus lactis*; MPL-TDM=**monophosphoryl-lipid A+trehalose dicorynomycolate; PBS=phosphate buffered solution; MBP=maltose-binding protein; IL=interleukin; Ig=immunoglobulin;** IFN-𝛾=interferon gamma; TNF-𝛼=tumor necrosis factor alpha; CFU=colony forming unit; PBMC=peripheral blood mononuclear cell; TTFC=tetanus toxin fragment C; TT=tetanus toxin; BCG=Bacillus Calmette-Guérin; MLD=minimal lethal dose; BAL=bronchoalveolar lavage; PADRE=pan allelic DR epitope; GLA-SE=glucopyranosyl lipid A in stable emulsion (TLR4 agonist); SAE=serious adverse event; AE=adverse event; PZQ=praziquantel.

Note: References were obtained through systematic search in PubMed without restrictions in language and time, including a reference search among the publications included in this review, and at the U.S. National Library of Medicine for clinical trial; the last searches were performed on April 25, 2021.
