# Supplementary material for: Recent Advances and Methodological Considerations on Vaccine Candidates for Human Schistosomiasis
Source: Front Trop Dis. Author manuscript; Available in PMC 2024 Sep 13. (PMC11392908; doi:10.3389/fitd.2021.719369)
Supplement: Table 3 [file NIHMS2017111-supplement-Table_3.docx]

**S3 Table.** Pre-clinical development of Sm-p80

| **Formulation** | **Design** | **Endpoints** | **Reference** |
| --- | --- | --- | --- |
| **Antigen:** rSm-p80 (expression system: *E. coli* BL21 (DE3))  **Adjuvant:** GLA-SE | **Experimental model:** baboons (*Papio anubis*)  **Administration:** immunization: i.m.; challenge: s.c.  **Immunization VG:** 250ug rSM-p80+50ug GLA-SE followed by 3 boosters of 250ug rSM-p80+50ug GLA-SE on day 28, 56 and 84 (CG: 50ug GLA-SE); challenge with 1000 *S. mansoni* cercariae on day 112; death on day 168 | **Worm reduction:** 66% total; 93% female worms  **Egg reduction:** 91% liver, 87% small intestine, 91% large intestine; 35-fold fecal excretion; 82% hatching  **Gene expression/Immunogenicity:** total IgG increasing with each booster, IgG1, IgA and IgM; 849 DEGs at vaccination, 1253 DEGs at necropsy and 149 DEGs common at both time points with upregulation of immune system-related pathways, innate and Th1-biased immune responses differently expressed in PBMCs, lymph node cells and splenocytes | Zhang W, et al., 2018  [138] |
| **Antigen:** rSm-p80 (expression system: *E. coli* BL21 (DE3))  **Adjuvant:** GLA-Alum | **Experimental model:** C57BL/6 mice, baboons (*Papio anubis*)  **Administration:** immunization: i.m.; challenge: s.c., axillary  **Immunization VG1** (mice)**:** 25ug rSm-p80 + 5ug GLA-Alum followed by 2 boosters of 25ug rSm-p80 + 5ug GLA-Alum on day 28 and 56 (CG: 5ug GLA-Alum); challenge with 150 *S. mansoni* cercariae on day 84 (s.c.); death on day 126  **Immunization VG2** (baboons)**:** 250ug rSm-p80 + 50ug GLA-Alum followed by 2 boosters of 250ug rSm-p80 + 50ug GLA-Alum on day 28 and 56 (CG: 50ug GLA-Alum); challenge with 1000 *S. mansoni* cercariae on day 84 (axillary); death on day 140 | **Worm reduction:** 43% (60% male and 40% female recovery) in VG1; 39% (49% male and 51% female recovery) in VG2  **Egg reduction:**  38% intestinal tissue eggs in VG1; 38% intestinal tissue eggs in VG2  **Immunogenicity:** total IgG, IgG1, IgG2A, IgG2B, and IgA, but weak IgM and IgG3 in VG1; total IgG, IgG1, IgG2A, IgG2B, IgG3, IgM and IgA in VG2; splenocytes, PBMCs and lymph node cells proliferated for IFN-𝛾, IL-2 and IL-10 (splenocytes) in VG1-2; IL-4 and IL-17 in VG1; IL-21 in VG2 | Zhang W, et al., 2018  [125] |
| **Antigen:** rSm-p80 (expression system: *E. coli* BL21 (DE3))  **Adjuvant:** GLA-SE | **Experimental model:** baboons (*Papio anubis*)  **Administration:** challenge: axillary  **Immunization:** 250ug rSm-p80 + 50ug GLA-SE followed by 2 boosters of 250ug rSm-p80 + 50ug GLA-SE on day 28 and 56 (CG: 50ug GLA-SE); challenge with 1000 *S. mansoni* cercariae (NMRI strain) on day 84; death on day 140 | **Egg reduction:** 68% liver; 86% hatching (4.33 ± 1.64 hatched eggs/gram; 8.59% ± 3.72 hatching rate; 52.53 ± 3.80 matured eggs/gram)  **Histopathology:** soft texture with few/small granulomas and necrosis; 39.93 ± 2.67 mm granuloma size; 237,759.7 mm^2^ cumulative granuloma area  **Immunogenicity:** total IgG | Le L, et al.,  2018  [143] |
| **Antigen:** rSm-p80 (expression system: *E. coli* BL21 (DE3))  **Adjuvant:** CpG-ODN | **Experimental model:** baboons (*Papio anubis*)  **Administration:** immunization: i.m.  **Trickle infection/treatment:** challenge with 200 *S. mansoni* cercariae followed by 4 booster challenges of 200 *S. mansoni* cercariae on day 7, 14, 21 and 28; treatment with PZQ 60mg/kg on day 91 followed by 2 booster treatments on day 119 and 175  **Immunization:** 250ug rSm-p80+250ug CpG-ODN on day 189 followed by 2 boosters of 250ug rSm-p80+250ug CpG-ODN on day 217 and 245 (CG: 250ug CpG-ODN); bolus challenge with 1000 *S. mansoni* cercariae on day 273; death on day 329 | **Worm reduction:** 14%  **Egg reduction:** 38% liver (50% matured, 60% hatched), 72% small intestine (53% matured, 49% hatched), 49% large intestine (88% matured, 82% hatched); 49% cumulative tissue  **Gene expression/Immunogenicity:** total IgG increase following PZQ and steady increase following immunization; trickle infection 2360 DEGs, chronic infection 1627 DEGs, PZQ 165 DEGs, and 99 DEGs common at these time points; immunization 254 DEGs, bolus challenge/necropsy 145 DEGs and 14 DEGs common at these time points | Siddiqui AJ, et al., 2018  [144] |
| **Antigen:** rSm-p80, Sm-p80/VR1020 (expression system: *E. coli* BL21 (DE3))  **Adjuvant:** GLA-SE, CpG-ODN10104 | **Experimental model:** C57BL/6 mice; hamsters  **Administration:** immunization: i.m.  **Immunization VG1** (mice)**:** 25ug rSm-p80+5ug GLA-SE followed by 2 boosters of 25ug rSm-p80+5ug GLA-SE on day 28 and 56 (CG: 5ug GLA-SE); challenge with 40 *S. japonicum* cercariae (Chinese strain) on day 84 (cover slip method); death on day 133  **Immunization VG2** (hamsters)**:** 400ug Sm-p80/VR1020+100ug CpG-ODN10104 and 100ug rSm-p80+100ug CpG-ODN10104 (CG: 100ug VR1020+100ug CpG-ODN10104); challenge with 2000 *S. haematobium* cercariae on day 28 (abdomen); death on day 133 | **Worm reduction:** 47% in VG1; 27% in VG2  **Egg reduction:** 5% liver, 40% (trial 1) intestine in VG1 (liver); 0% (liver, intestine, urinary bladder) in VG2  **Immunogenicity:** total IgG in VGs1-2; IgG1, IgG2A, IgG2B, IgG3 IgA and IgM in VG1; splenocytes proliferated for IFN-𝛾, TNF-𝛼, IL-2, IL-4 and IL-12 in VGs1-2 | Molehin AJ, et al., 2017  [124] |
| **Antigen:** rSm-p80, Sm-p80-VR1020, Sm-p80-pcDNA3 (expression system: *E. coli* BL21 (DE3))  **Adjuvant:** alum, CpG-ODN, GLA-SE | **Experimental model:** C57BL/6 mice; baboons (*Papio anubis*)  **Administration:** immunization: i.m.; challenge: s.c. (mice), p.c. (baboons)  **Immunization VG1:** 100ug Sm-p80-VR1020 followed by 2 boosters of 100ug Sm-p80-VR1020 on day 28 and 56 (CG: 100ug VR1020); challenge with 150 *S. mansoni* cercariae on day 84; death on day 140  **Immunization VG2:** 100ug Sm-p80-pcDNA3+50ug CpG-ODN and 25ug rSm-p80+50ug CpG-ODN followed by 2 boosters of 100ug Sm-p80-pcDNA3+50ug CpG-ODN and 25ug rSm-p80+50ug CpG-ODN on day 28 and 56 (CG: 100ug pcDNA3+50ug CpG-ODN); challenge with 150 *S. mansoni* cercariae on day 84; death on day 140  **Immunization VG3:** 25ug rSm-p80+150ug alum followed by 2 boosters of 25ug rSm-p80+150ug alum on day 28 and 56 (CG: 150ug alum); challenge with 150 *S. mansoni* cercariae on day 84; death on day 140  **Immunization VG4:** 25ug rSm-p80+50ug CpG-ODN followed by 2 boosters of 25ug rSm-p80+50ug CpG-ODN on day 28 and 56 (CG: 50ug CpG-ODN); challenge with 150 *S. mansoni* cercariae on day 84; death on day 140  **Immunization VG5:** 25ug rSm-p80+5ug GLA-SE followed by 2 boosters of 25ug rSm-p80+5ug GLA-SE on day 28 and 56 (CG: 5ug GLA-SE); challenge with 150 *S. mansoni* cercariae on day 84; death on day 140  **Immunization VG6:** 25ug rSm-p80+50ug CpG-ODN10104 followed (CG: 50ug CpG-ODN2137); death on day 1, 2, 7 and 21  **Immunization VG7** (baboons)**:** 250ug rSm-p80+250ug ODN followed by 2 boosters of 250ug rSm-p80+250ug ODN on day 28 and 56 (CG: 250ug ODN); challenge with 1000 *S. mansoni* cercariae on day 84; death on day 140 | **Gene expression/Immunogenicity:** 83 DEGs expressed in murine VGs1-5 (59 DEGs in control challenged mice (VGs1-5) related to Ig-gene rearrangement (decrease in IghD, increase in IghA, IghG1, IghG2) and 24 DEGs in non-challenged mice (VG6) related to IRFs, MHC I/II, complement and transcriptional factors, proteins, enzymes, and canonical pathways); shift from innate to adaptive immune response 21 days post vaccination in VG6; activation and proliferation of innate inflammatory elements (S100A family, IRF7, IL17, IL6, INFA) and adaptive cellular Th1 cells, CD8 T-cells, humoral responses with B-cell differentiation in VG7 | Rojo JU, et al., 2017 |
| **Antigen:** rSm-p80, Sm-p80-VR1020 (expression system: *E. coli* BL21 (DE3))  **Adjuvant:** GLA-SE, alum, CpG-ODN10104 | **Experimental model:** baboons (*Papio anubis*) chronically infected with 1000 *S. mansoni* cercariae (trickle infection)  **Administration:** immunization: i.m.  **Immunization VG1:** 250ug rSm-p80+50ug GLA-SE on day 112 followed by 2 boosters of 250ug rSm-p80+50ug GLA-SE on day 140 and 168 (CG: 50ug GLA-SE); death on day 216  **Immunization VG2:** 500ug Sm-p80-VR1020 on day 49 followed by 2 boosters of 250ug rSm-p80+1250ug alum on day 77 and 147 (CG: 500ug VR1020, 1250ug alum); death on day 157  **Immunization VG3:** 500ug Sm-p80-VR1020 on day 147 followed by 2 boosters of 250ug rSm-p80+250ug CpG-ODN10104 on day 175 and 217 (CG: 500ug VR1020, 250ug CpG-ODN); challenge with 1000 *S. mansoni* cercariae and *Trichuris* on day 189; death on day 266 | **Worm reduction:** 36% in VG1; 10% in VG2; 23% in VG3  **Egg reduction:** 54% tissue, 33% feces in VG1; 10% tissue, 15% feces in VG2; 57% tissue, 13% feces in VG3  **Immunogenicity:** total IgG and IgM in VGs1-3; no IgG2, IgG3, IgG4 and IgA (weak) in VG1; IgG2, IgG4 and IgA in VG2; no difference to controls for IgG2, IgG3 and IgG4 but IgA (weak) in VG2; PBMCs, splenocytes and lymph node cells proliferated for IFN-𝛾, IL-2, IL-12a, IL-12b, TNF-𝛼, IL-17, IL-21 and IL-22 in VG1; mixed proliferation for Th17 cytokines in VG2; IFN-𝛾, IL-2, IL-12a, IL-12b, IL-17 and IL-23 in VG3 | Karmakar S, et al., 2014  [141] |
| **Antigen:** rSm-p80 (expression system: *E. coli* BL21 (DE3))  **Adjuvant:** GLA-SE | **Experimental model:** golden Syrian hamsters; baboons (*Papio anubis*)  **Immunization VG1** (hamsters)**:** 100ug rSm-p80+10ug GLA-SE followed by 2 boosters of 100ug rSm-p80+10ug GLA-SE on day 28 and 56 (CG: 10ug GLA-SE); challenge with 200 *S. haematobium* cercariae (Egypt strain) on day 154; death on day 196  **Immunization VG2** (baboons)**:** 250ug rSm-p80+50ug GLA-SE followed by 2 boosters of 250ug rSm-p80+50ug GLA-SE on day 28 and 56 (CG: 50ug GLA-SE); challenge with 1000 *S. haematobium* cercariae (Egypt strain) on day 154; death on day 217 | **Worm reduction:** 48% worm in VG1; 25% in VG2  **Egg reduction:** 66% and 63% in liver and intestine, respectively and no eggs in urinary bladder in VG1; no reduction in liver and intestine, 64% in urinary bladder and 40% an 53% in feces and urine in VG2  **Immunogenicity:** total IgG in VG1; total IgG, IgG1, IgA, and IgM but no IgG2, IgG3 and IgG4 in VG2; splenocytes and PBMCs proliferated for IFN-𝛾, IL-4 and IL-13 in VG1; lymph node cells proliferated for IFN-𝛾, IL-12b, IL-1a and IL-8 in VG2 | Karmakar S., et al., 2014 [127] |
| **Antigen:** rSm-p80, Sm-p80-pcDNA3  **Adjuvant:** GLA-SE, IL-2 | **Experimental model:** C57BL/6 mice, baboons (*Papio anubis*)  **Administration:** immunization: i.m.  **Immunization VG1** (mice)**:** 25ug rSm-p80+5ug GLA-SE followed by eat least 1 booster of 25ug rSm-p80+5ug GLA-SE on day 28 and 2^nd^ booster of 25ug rSm-p80+5ug GLA-SE on day 56 in some mice (CG: 5ug GLA-SE); no challenge or challenge with 150 *S. mansoni* cercariae (NMRI strain) on day 56, 84, 98 or 140; no death or death on day 98, 126, 140 or 186  **Immunization VG2** (baboons)**:** Sm-p80-pcDNA3 or Sm-p80-pcDNA3+pORF-hiL-2; Sm-p80-VR1020 (pregnant baboon); follow-up 5-8 years | **Worm reduction:** 42% with 2 boosters and 36%, 32% and 15% with 1 booster in VG1  **Immunogenicity:** total IgG, IgG1, IgG2A, IgG2B, IgG3, IgM, IgA differently regulated depending on booster frequency in VG1; total IgG detectable between 5-8 years in VG2; transfer of total IgG through placenta/colostrum/ lactation up to 6 weeks postnatal in VG2 | Zhang W, et al., 2014  [140] |
| **Antigen:** rSm-p80, Sm-p80-VR1020 (expression system: *E. coli* BL21 (DE3))  **Adjuvant:** CpG-ODN, ODN10104 | **Experimental model:** C57BL/6 mice  **Administration:** immunization: i.m.  **Immunization:** 100ug Sm-p80-VR1020+50ug CpG-ODN and 25ug rSm-p80+50ug ODN10104 (CG: VR1020, ODN2137); challenge with 150 *S. mansoni* cercariae (NMRI strain) on day 28; death on day 70 | **Worm reduction:** 34 and 57% in repeated testing  **Egg reduction:** 66% and 34% in repeated testing  **Immunogenicity:** total IgG, IgG1, IgG2A, IgG2B, IgG3, IgM and IgA; splenocyte proliferation for IFN-𝛾, IL-2, IL-12a, IL-17, IL-1a, IL-1b, IL-4 and IL-13 | Le L, et al., 2014  [142] |
| **Antigen:** rSm-p80  **Adjuvant:** CpG-ODN10104 | **Experimental model:** C57BL/6 mice, B6-129S4-C3tm1crr/J (C3 deficient)  **Administration:** immunization: i.m.; challenge: s.c.  **Immunization VG1** (C57BL/6 mice)**:** 25ug rSm-p80+50ug CpG-ODN10104 followed by 2 boosters of 25ug rSm-p80+50ug CpG-ODN10104 on day 28 and 56 (CG: ODN2137); challenge with 150 *S. mansoni* cercariae (NMRI strain) on day 84; death on day 126  **Immunization VG2** (B6-129S4-C3tm1crr/J)**:** 25ug rSm-p80+50ug CpG-ODN10104 followed by 2 boosters of 25ug rSm-p80+50ug CpG-ODN10104 on day 28 and 56 (CG: ODN2137); challenge with 150 *S. mansoni* cercariae (NMRI strain) on day 84; death on day 126 | **Worm reduction:** 53% in VG1; 34% in VG2  **Egg reduction:** 15-25-fold lower in VG2 than VG1 (liver, intestine)  **Immunogenicity:** in vitro schistosomula killing larger with exogeneous complement in mouse/baboon sera than heat-inactivated sera than sera with CVF; total IgG, IgG1, IgG2A, IgG2B, IgG3 and IgM in VGs1-2; IL-2, IL-21 and IFN-𝛾 in VGs1-2; IL-17, TNF-𝛼 and IL-5 in VG1; IL-4 and IL-8 in VG2 | Karmakar S, et al., 2014  [121] |
| **Antigen:** rSm-p80  **Adjuvant:** CpG-ODN10104 | **Experimental model:** C57BL/6 mice  **Immunization:** 25ug rSm-p80+50ug CpG-motif ODN10104 followed by 2 boosters of 25ug rSm-p80+50ug CpG-ODN10104 each on day 28 and 56 (CG: CpG-ODN); death on day 56/57 | **Cytotoxicity: i**mmune killing of schistosomula through activated macrophages and lymphocytes (CD3+CD4+T-cells) in immune sera and lung lavage/lung cells related to toxic nitric oxoide production | Torben W, et al., 2012  [139] |
| **Antigen:** rSm-p80, Sm-p80-VR1020  **Adjuvant:** CpG-ODN, R848 | **Experimental model:** baboons (*Papio anubis*)  **Immunization VG1:** 250ug rSm-p80+250ug ODN10104 followed by 2 boosters of 250ug rSm-p80+250ug ODN10104 each on day 28 and 56 (CG: ODN10104); challenge with 1000 *S. mansoni* cercariae on day 84; death on day 140/147  **Immunization VG2:** 250ug rSm-p80+50ug R848 followed by 2 boosters of 250ug rSm-p80+50ug R848 each on day 28 and 56 (CG: BA+R848); challenge with 150 *S. mansoni* cercariae on day 84; death on day 140/147  **Immunization VG3:** 500ug Sm-p80-VR1020 followed by 2 boosters of 250ug rSm-p80+250ug ODN10104 each on day 28 and 56 (CG: VR1020, ODN); challenge with 1000 *S. mansoni* cercariae on day 84; death on day 140/147  **Immunization VG4:** 500ug Sm-p80-VR1020 followed by 2 boosters of 250ug rSm-p80+50ug R848 each on day 28 and 56 (CG: VR1020, BA+R848); challenge with 100 *S. mansoni* cercariae on day 84; death on day 140/147  **Correlate study:** Anti-Sm-p80 IgG and IgE (hypersensitivity) in uninfected adults from Nairobi, Kenya, and occupational hyper-exposed male adults and school children from Lake Victoria, Kenya | **Worm reduction:** 58% in VG1, 52% in VG2, 47% in VG3, 38% in VG4  **Immunogenicity:** total IgG, IgG1, IgG2, IgA and IgM, but no IgG3 and IgG4 in VGs1-4; PBMC, splenocyte and lymph node cells proliferation for IFN-𝛾 and IL-2, but unremarkable for IL-4 and IL-10 in VGs1-4; IL-21, IL-1b, IL-22 in VGs1-2; IL-12 and IL-6 in VGs3-4; hyper-exposed males with elevated anti-Sm-p80 IgG; school children with weak anti-Sm-p80 IgG; no anti-Sm-p80 IgE | Ahmad G, et al., 2011  [40] |
| **Antigen:** Sm-p80-VR1020, rSm-p80 (expression system: *E. coli* BL21 (DE3); CHO and COS-7 cells)  **Adjuvant:** alum | **Experimental model:** C57BL/6 mice  **Administration:** challenge: s.c.  **Immunization VG1:** 100ug Sm-p80-VR1020 followed by 2 boosters of 25ug rSm-p80+150ug alum each on day 28 and 56 (CG: VR1020, alum); challenge with 150 *S. mansoni* cercariae on day 84; death on day 126  **Immunization VG2:** 25ug rSm-p80+150ug alum followed by 2 boosters of 25ug rSm-p80+150ug alum each on day 28 and 56 (CG: alum); challenge with 150 *S. mansoni* cercariae on day 84; death on day 126 | **Worm reduction:** 61% in VG1; 55% in VG2  **Egg reduction:** 23% in VG1; 21% in VG2  **Immunogenicity:** total IgG, IgG1, IgG2A, IgG2B, IgG3 and IgM (moderate) in VGs1-2; splenocyte proliferation rate 26% in VG1 and 21% in VG2 compared to controls; IFN-𝛾, IL-2, IL-4 and IL-10 (insignificant), IL-3, IL-5, IL6, MIP-2, IL-9 and IL-12 in VGs1-2 | Zhang W, et al., 2011  [136] |
| **Antigen:** Sm-p80-pcDNA3  **Adjuvant:** CpG-motif ODN | **Experimental model:** C57BL/6 mice (naïve, antibody knock-out)  **Administration:** immunization: i.v.  **Immunization VG1** (homologous transfer, naïve mice)**:** 2 boosters of 100ul of sera from mice immunized with Sm-p80-pcDNA3 each on day -6 and -2 (CG: pcDNA3 sera); challenged with 150 S*. mansoni* cercariae on day 0; death on day 42  **Immunization VG2** (heterologous transfer, naïve mice)**:** 2 boosters of 100ug of purified IgG of sera of baboons immunized with Sm-p80-pcDNA3 each on day -6 and -2 (CG: IgG pcDNA3 sera); challenged with 150 *S. mansoni* cercariae on day 0; death on day 42  **Immunization VG3** (naïve and knock-out mice)**:** 25ug rSm-p80+50ug CpG-motif ODN followed by 2 boosters of 25ug rSm-p80+50ug CpG-motif ODN each on day 28 and 56 (CG: CpG-motif ODN); challenge with 150 *S. mansoni* cercariae on day 84; death on day 126 | **Worm reduction:** 31% in VG1; 45% in VG2; 18% (knock-out) and 63% (naïve) in VG3  **Egg reduction:** 59% in VG1; 90% in VG2; 36% (knock-out) and 47% (naïve) in VG3 (liver, intestine)  **Immunogenicity:** total IgG, IgG1, IgG2A, IgG2B, IgG3 and IgM, but no IgA in VG1-2; splenocyte proliferation for IFN-𝛾 and IL-2 but moderate IL-4 and IL-10 in VG1 (IL-6, TNF-𝛼) and VG2 (IL-6, IL-12a, IL-17, TGF-𝛽1); TGF-𝛽1/2, TNF-𝛼, IFN-𝛾, IL-2, IL-4, IL-5, IL-12a/b, IL-16-18, IL-20, IL-22, IL23 | Torben W, et al., 2011  [137] |
| **Antigen:** Sm-p80-pcDNA3, rSm-p80  **Adjuvant:** R848 | **Experimental model:** C57BL/6 mice  **Administration:** immunization: i.m.; challenge: s.c.  **Immunization VG1:** 100ug Sm-p80-pcDNA3 followed by 2 boosters of 25ug rSm-p80+10ug R848 each on day 28 and 56 (CG: pcDNA3, R848); challenge with 150 *S. mansoni* cercariae (Puerto Rico strain) on day 84; death on day 130  **Immunization VG2:** 25ug rSm-p80+10ug R848 followed by 2 boosters of 25ug rSm-p80+10ug R848 each on day 28 and 56 (CG: R848); challenge with 150 *S. mansoni* cercariae (Puerto Rico strain) on day 84; death on day 130 | **Worm reduction:** 49% in VG1; 50% in VG2  **Egg reduction:** 30% in VG1; 16% in VG2 (liver, intestine)  **Immunogenicity:** total IgG, IgG1, IgG2A, IgG2B, IgG3 (high), IgM and IgA (high) in VGs1-2; splenocyte proliferation for IFN-𝛾 (high) and IL-2 (high), but moderate IL-4 and IL-10 in VG1 (IL-3, IL-12a, IL-15) and VG2 (IL-6, IL-16) | Ahmad G, et al., 2010  [132] |
| **Antigen:** Sm-p80-VR1020 (expression system: CHO and COS-7 cells) | **Experimental model:** baboons (*Papio anubis*)  **Administration:** immunization: i.m.  **Immunization:** 500ug Sm-p80-VR1020 followed by 3 boosters of 500ug Sm-p80-VR1020 each on day 28, 56 and 84 (CG: VR1020); challenge with 1000 *S. mansoni* cercariae on day 112; death on day 168 | **Worm reduction:** 46% (44% males, 19% females, 19% paired worms, no immature worms)  **Egg reduction:** 28% (liver, intestine)  **Immunogenicity:** total IgG, IgG1, IgG2, IgA (moderate) and IgM (moderate), but no IgG3, IgG4 and IgE; 56% PBMC and 38% splenocyte proliferation for IFN-𝛾, IL-2 and negligible IL-4 and IL-10  **Toxicity/Safety:** No abnormal animal behavior/clinical observations; well tolerated | Zhang W, et al., 2010  [135] |
| **Antigen:** Sm-p80-VR1020 (expression system: CHO K1 and COS-7 cells) | **Experimental model:** C57BL/6 mice  **Administration:** challenge: s.c.  **Immunization:** 100ug Sm-p80-VR1020 followed by 3 boosters of 100ug Sm-p80-VR1020 each on day 28, 56 and 84 (CG: VR1020); challenge with 150 *S. mansoni* cercariae (Puerto Rico strain) on day 112; death on day 158 | **Worm reduction:** 47%  **Immunogenicity:** total IgG, IgG1, IgG2A, IgG2B (doubled IgG2A), IgG3 (weak), IgM (weak) and IgA (weak); splenocytes proliferated for IFN-𝛾 and IL-2 (IL-1b, IL-3, IL-5, IL-6, IL-8, IL-11, IL-12, IL-15-18, IL-21, TGF-𝛽, TNF-𝛼) but no IL-4 and IL-10 | Zhang W, et al., 2010  [120] |
| **Antigen:** Sm-p80-pcDNA3 (expression system: CHO cells) | **Experimental model:** C57BL/6 mice  **Administration:** immunization: i.m.; challenge: s.c.  **Immunization:** 100ug Sm-p80-pcDNA3 followed by 3 boosters of 100ug Sm-p80-pcDNA3 each on day 28, 56 and 84 (CG: pcDNA3); challenge with 150 *S. mansoni* cercariae (Puerto Rico strain) on day 112; death on day 158 | **Worm reduction:** 59%  **Egg reduction:** 84%  **Immunogenicity:** total IgG, IgG1, IgG2A, IgG2B and IgG3; splenocyte proliferation of IFN-𝛾 and IL-2, but no IL-4 and IL-10 | Ahmad G, et al., 2009  [131] |
| **Antigen:** Sm-p80-pcDNA3, rSm-p80 (expression system: *E. coli* BL21 (DE3))  **Adjuvant:** CpG-ODN | **Experimental model:** C57BL/6 mice  **Administration:** DNA prime immunization: i.m.; protein boost immunization: s.c.  **Immunization VG1:** 100ug Sm-p80-pcDNA3 followed by 2 boosters of 25ug rSm-p80+50ugODN-10104 each on day 28 and 56 (CG: pcDNA3, ODN-2137); challenge with 150 *S. mansoni* cercariae (Puerto Rico strain) on day 84; death on day 126  **Immunization VG2:** 25ug rSm-p80+50ugODN-10104 followed by 2 boosters of 25ug rSm-p80+50ugODN-10104 each on day 28 and 56 (CG: ODN-2137); challenge with 150 *S. mansoni* cercariae (Puerto Rico strain) on day 84; death on day 126 | **Worm reduction:** 57% in VG1 (44% males, 56% females); 70% in VG2 (43% males, 57% females)  **Egg reduction:** 71% in VG1 with 65% anti-fecundity effect; 75% in VG2 with 77% anti-fecundity effect  **Immunogenicity:** total IgG, IgG1, IgG2A, IgG2B, IgG3, IgA and IgM in VGs1-2; IFN-𝛾, splenocyte proliferation of IL-2 and IL-17 in VG1, and IFN-𝛾, IL-2, IL-12, IL-22 and IL-8 in VG2 | Ahmad G, et al., 2009  [30] |
| **Antigen:** Sm-p80-pcDNA3 (expression system: CHO-K1 and COS-7 cells) | **Experimental model:** baboons (*Papio anubis*)  **Administration:** immunization: i.m.; challenge: s.c.  **Immunization:** 500ug Sm-p80-pcDNA3 followed by 3 boosters of 500ug Sm-p80-pcDNA3 each on day 28, 56 and 84 (CG: pcDNA3); challenge with 1000 *S. mansoni* cercariae (Puerto Rico strain) on day 112; death on day 168 | **Worm reduction:** 38% (30% males, 14% females, 50% paired worms, 6% immature worms)  **Egg reduction:** 32% (liver, intestine)  **Immunogenicity:** total IgG, IgG1, IgG2, IgM and IgA (weak), but no IgG3, IgG4 and IgE; PBMC proliferation of IFN-𝛾 and IL-2 but negligible of IL-4 and IL-10  **Toxicity/Safety:** No abnormal animal behavior/clinical observations; well tolerated | Ahmad G, et al., 2009  [134] |
| **Antigen:** Sm-p80-pcDNA3  **Adjuvant:** IL-2 | **Experimental model:** baboons (*Papio anubis*)  **Administration:** i.m. immunization  **Immunization VG1:** 500ug Sm-p80-pcDNA3 followed by 3 boosters of 500ug Sm-p80-pcDNA3 each on day 28, 56 and 84 (CG: pcDNA3); death on day 112  **Immunization VG2:** 500ug Sm-p80-pcDNA3+500ug pORF-hIL-2 followed by 3 boosters of 500ug Sm-p80-pcDNA3+500ug pORF-hIL-2 each on day 28, 56 and 84 (CG: pcDNA3); death on day 112 | **Immunogenicity:** IgG1/IgG2 <1 in VGs1-2; total IgG (moderate), but no IgG3 and IgG4 in VGs1-2; 21-34% and 21-24% complement-dependent killing in VGs1-2, respectively  **Toxicity/Safety:** No abnormal animal behavior/clinical observations; well tolerated | Siddiqui AA, et al., 2005  [133] |
| **Antigen:** Sm-p80-pcDNA3  **Adjuvant:** GM-CSF, IL-4, IL-2, IL-12 | **Experimental model:** C57BL/6 mice  **Administration:** immunization: i.m.; challenge: s.c.  **Immunization VG1:** 100ug Sm-p80-pcDNA3 followed by 2 boosters of 100ug Sm-p80-pcDNA3 each on day 28 and 56 (CG: pcDNA3); challenge with 150 *S. mansoni* cercariae on day 84; death on day 126  **Immunization VG2:** 100ug Sm-p80-pcDNA3+100ug pORF-mGM-CSF followed by 2 boosters of 100ug Sm-p80-pcDNA3+100ug pORF-mGM-CSF each on day 28 and 56 (CG: pcDNA3); challenge with 150 *S. mansoni* cercariae on day 84; death on day 126  **Immunization VG3:** 100ug Sm-p80-pcDNA3+100ug pORF-mIL-4 followed by 2 boosters of 100ug Sm-p80-pcDNA3+100ug pORF-mIL-4 each on day 28 and 56 (CG: pcDNA3); challenge with 150 *S. mansoni* cercariae on day 84; death on day 126  **Immunization VG4:** 100ug Sm-p80-pcDNA3+100ug pORF-mIL-12 followed by 2 boosters of 100ug Sm-p80-pcDNA3+100ug pORF-mIL-12 each on day 28 and 56 (CG: pcDNA3); challenge with 150 *S. mansoni* cercariae on day 84; death on day 126  **Immunization VG5:** 100ug Sm-p80-pcDNA3+100ug pORF-mIL-2 followed by 2 boosters of 100ug Sm-p80-pcDNA3+100ug pORF-mIL-2 each on day 28 and 56 (CG: pcDNA3); challenge with 150 *S. mansoni* cercariae on day 84; death on day 126 | **Worm reduction:** 39% in VG1; 44% in VG2, 42% in VG3; 45% in VG4, 57% in VG5  **Immunogenicity:** splenocyte proliferation and IFN-𝛾 production in VG1 < VG3 < VG2 < VG4 < VG5; total IgG, IgG2A and IgG2B in VG1; IgG1 and IgG3 in VG3; total IgG, IgG2A, and IgG2B in VG5; total IgG and IgG2A in VG4 | Siddiqui AA, et al., 2005  [130] |
| **Antigen:** Sm-p80-pcDNA3 (expression system: CHO cells)  **Adjuvant:** IL-4, GM-CSF | **Experimental model:** C57BL/6 mice  **Administration:** immunization: i.m.; challenge: s.c.  **Immunization VG1:** 100ug Sm-p80-pcDNA3 followed by 2 boosters of 100ug Sm-p80-pcDNA3 each on day 28 and 56 (CG: pcDNA3); challenge with 150 *S. mansoni* cercariae (Puerto Rico strain) on day 84; death on day 126  **Immunization VG2:** 100ug Sm-p80-pcDNA3+100ug pORF-mGM-CSF followed by 2 boosters of 100ug Sm-p80-pcDNA3+100ug pORF-mGM-CSF each on day 28 and 56 (CG: pcDNA3); challenge with 150 *S. mansoni* cercariae (Puerto Rico strain) on day 84; death on day 126  **Immunization VG3:** 100ug Sm-p80-pcDNA3+100ug pORF-mIL-4 followed by 2 boosters of 100ug Sm-p80-pcDNA3+100ug pORF-mIL-4 each on day 28 and 56 (CG: pcDNA3); challenge with 150 *S. mansoni* cercariae (Puerto Rico strain) on day 84; death on day 126 | **Worm reduction:** 39% in VG1; 44% in VG2; 42% in VG3  **Immunogenicity:** total IgG, IgG2A (moderate), IgG2B, IgG3 and IgG1 in VGs1-3 though IgG1 unremarkable in VG1; no IgM, IgA and IgE in VGs1-3 | Siddiqui AA., et al., 2003  [118] |
| **Antigen:** Sm-p80-pcDNA3 (expression system: CHO cells)  **Adjuvant:** IL-12, IL-2 | **Experimental model:** C57BL/6 mice  **Administration:** immunization: i.m.; challenge: s.c.  **Immunization VG1:** 100ug Sm-p80-pcDNA3 followed by 2 boosters of 100ug Sm-p80-pcDNA3 each on day 28 and 56 (CG: pcDNA3); challenge with 150 *S. mansoni* cercariae (Puerto Rico strain) on day 84; death on day 126  **Immunization VG2:** 100ug Sm-p80-pcDNA3+100ug pORF-mIL-2 followed by 2 boosters of 100ug Sm-p80-pcDNA3+100ug pORF-mIL-2 each on day 28 and 56 (CG: pcDNA3); challenge with 150 *S. mansoni* cercariae (Puerto Rico strain) on day 84; death on day 126  **Immunization VG3:** 100ug Sm-p80-pcDNA3+100ug pORF-mIL-12 followed by 2 boosters of 100ug Sm-p80-pcDNA3+100ug pORF-mIL-12 each on day 28 and 56 (CG: pcDNA3); challenge with 150 *S. mansoni* cercariae (Puerto Rico strain) on day 84; death on day 126 | **Worm reduction:** 39% in VG1; 57% in VG2; 45% in VG3  **Immunogenicity:** total IgG, IgG1 (unremarkable) and IgG3 (moderate), but no IgM, IgA and IgE in VGs1-3; IgG2A and IgG2B in VGs2-3 | Siddiqui AA, et al., 2003  [53] |
| **Antigen:** p80WT (=wild-type), p80M (=mutant, substitution of cysteine to serine) (expression system: *E.coli* DH5a) | **Experimental model:** C57BL/6 mice  **Administration:** immunization; i.n., s.c. or gene gun abdominal; challenge: s.c.  **Immunization VG1 (s.c.):** 10^7^pfu RVV+p80sWT or p80sM or p80asM followed by 2 boosters each on day 14 and 42; challenge with 300 *S. mansoni* cercariae (Puerto Rico strain) on day 77; death on day 116  **Immunization VG2 (i.n.):** 10^7^pfu RVV+p80sWT or p80sM or p80asM followed by 2 boosters each on day 14 and 42; challenge with 300 *S. mansoni* cercariae (Puerto Rico strain) on day 77; death on day 116  **Immunization VG3 (s.c.):** 10^7^pfu RVV+p80sWT or p80sM or p80asM followed by 3 boosters each on day 14, 28 and 98; challenge with 300 *S. mansoni* cercariae (Puerto Rico strain) on day 147; death on day 182  **Immunization VG4:** 0.5ug WRG+p80WT or p80M followed by 10^7^pfu RVV+p80sWT or p80sM or p80asM on day 14 (helium-driven gene gun priming, s.c. boosting); challenge with 300 *S. mansoni* cercariae (Puerto Rico strain) on day 42; death on day 84  **Immunization VG5:** 0.5ug WRG+p80WT or p80M followed by 10^7^pfu RVV+p80sWT or p80sM or p80asM on day 14 (helium-driven gene gun priming, s.c. boosting); challenge with 300 *S. mansoni* cercariae (Puerto Rico strain) on day 70 prior to bleeding on day 49; death on day 116  **Immunization VG6:** 0.5ug WRG+p80WT or p80M followed by 2 boosters each on day 14 and 28 (helium-driven gene gun); challenge with 300 *S. mansoni* cercariae (Puerto Rico strain) on day 56; death on day 98  **Immunization VG7:** 0.5ug WRG+p80WT or p80M followed by 2 boosters each on day 14 and 42 (helium-driven gene gun); challenge with 300 *S. mansoni* cercariae (Puerto Rico strain) on day 70 following bleeding on day 63; death on day 112 | **Worm reduction:** no protection in VGs1-5; 60% in VG6  **Immunogenicity:** anti-p80WT and p80M total IgG, IgG1, IgG2A and IgG2B in VG2; anti-p80WT and p80M IgG1 and IgG2A in VG7 | Hota-Mitchell S, et al., 1999  [129] |
| **Antigen:** APM; CaBP-APM; CaBP-Sm-p80 (expression system: Baculovirus/Sf-9 cells)  **Adjuvant:** CFA; IFA; SAF-1 | **Experimental model:** C57BL/6 mice  **Administration:** immunization: s.c.; challenge: s.c.  **Immunization VG1:** 10ug APM+CFA/SAF-1 followed by 2 boosters of 10ug APM+IFA/SAF-1 each on day 21 and 35 (CG: CFA/IFA or SAF-1); challenge with 300 *S. mansoni* cercariae (Puerto Rico strain) on day 49; death on day 91  **Immunization VG2:** 50ug CaBP-APM+CFA/SAF-1 followed by 2 boosters of 25ug CaBP-APM+IFA/SAF-1 each on day 14 and 28 (CG: CFA/IFA or SAF-1); challenge with 300 *S. mansoni* cercariae (Puerto Rico strain) on day 42; death on day 84  **Immunization VG3:** 25ug CaBP-Sm-p80+CFA/SAF-1 followed by 2 boosters of 10ug CaBP-Sm-p80+IFA/SAF-1 each on day 14 and 28 (CG: CFA/IFA or SAF-1); challenge with 300 *S. mansoni* cercariae (Puerto Rico strain) on day 42; death on day 84 | **Worm reduction:** 34% with CFA and 40% with SAF-1 in VG1; 67% with CFA and 56% with SAF-1 in VG2; 39% with CFA and 29% with SAF-1 in VG3  **Immunogenicity:** 67% total antibody reactivity to recombinant baculovirus-produced Sm-p80 in chronically infected humans: 25% IgA, 17% IgM, 42% total IgG, 42% IgG1 and 8% IgG3, but no IgE, IgG2 and IgG4 | Hota-Mitchell S, et al., 1997  [126] |

Abbreviations: VG=vaccine group; CG=control group; s.c.=subcutaneous; i.m.=intramuscular; i.v.=intravenous; i.n.=intranasal; p.c.=percutaneous; IL=interleukin; Ig=immunoglobulin; IFN-𝛾 =interferon gamma; TNF-𝛼 =tumor necrosis factor alpha; RVV=recombinant vaccinia virus; Sf-9 cells=clonal isolate from *Spodoptera frugiperda* IPLB-Sf21-AE cells; CHO cells=Chinese hamster ovary cells; COS-7 cells=African green monkey kidney cells; PBMC=peripheral blood mononuclear cell; APM=apical plasma membrane; MIP=macrophage inflammatory protein; TGF-𝛽=transforming growth factor beta; CaBP-APM=calcium-binding protein; CFA=complete Freund’s adjuvant; IFA=incomplete Freund’s adjuvant; SAF-1=syntax adjuvant formulation; GM-CSF=granulocyte-macrophage colony-stimulating factor; CpG-ODN=oligodeoxynucleotides with unmethylated CpG dinucleotides (TLR9 agonist); R848=resiquimod (TLR7 agonist); alum=aluminum hydroxide; BA=baboon albumin; CVF=cobra venom factor; GLA-SE=glucopyranosyl lipid A in stable emulsion (TLR4 agonist)/glucopyranosyl lipid A formulated in a stable nano-emulsion of squalene oil-in-water (TLR4 agonist); DEG=differentially expressed genes; IRF=interferon regulatory factor; MHC=major histocompatibility complex; PZQ=praziquantel; *S. mansoni*/Sm=*Schistosoma mansoni*; *S. haematobium*=*Schistosoma haematobium*; *S. japonicum*=*Schistosoma japonicum*; *E.coli*=*Escherichia coli*.

Note: References were obtained through systematic search in PubMed without restrictions in language and time, including a reference search among the publications included in this review, and at the U.S. National Library of Medicine for clinical trials; the last searches were performed on April 25, 2021.
