# Supplementary material for: Recent Advances and Methodological Considerations on Vaccine Candidates for Human Schistosomiasis
Source: Front Trop Dis. Author manuscript; Available in PMC 2024 Sep 13. (PMC11392908; doi:10.3389/fitd.2021.719369)
Supplement: Table 1 [file NIHMS2017111-supplement-Table_1.docx]

**Table S1.** Pre-clinical and clinical development of Sm-TSP-2

| **Formulation** | **Design** | **Endpoints** | **Reference** |
| --- | --- | --- | --- |
| **Antigen:** rSm-TSP-2  **Adjuvant:** Aldydrogel, AP10-701 | **Experimental model:** humans (healthy adults 18-45 years from *S. mansoni* endemic area Uganda)  **Administration:** immunization: i.m.  *Phase I dose-escalation safety and immunogenicity study among healthy exposed adults*  **Immunization VG1:** 10ug rSm-TSP-2/Alhydrogel or 10ug rSm-TSP-2/Alhydrogel+AP10-701 or Engerix-B followed by 2 boosters of 10ug rSm-TSP-2/Alhydrogel or 10ug rSm-TSP-2/Alhydrogel+ AP10-701 or Engerix-B each on day 56 and 120  **Immunization VG2:** 30ug rSm-TSP-2/Alhydrogel or 30ug rSm-TSP-2/Alhydrogel+AP10-701 or Engerix-B followed by 2 boosters of 30ug rSm-TSP-2/Alhydrogel or 30ug rSm-TSP-2/Alhydrogel+ AP10-701 or Engerix-B each on day 56 and 120  **Immunization VG3:** 100ug rSm-TSP-2/Alhydrogel or 100ug rSm-TSP-2/Alhydrogel+AP10-701 or Engerix-B followed by 2 boosters of 100ug rSm-TSP-2/Alhydrogel or 100ug rSm-TSP-2/Alhydrogel+ AP10-701 or Engerix-B each on day 56 and 120  *Phase IIb trial on vaccine impact with S. mansoni and cross-species protection with S. haematobium*  **Immunization VG1:** rSm-TSP-2/Alhydrogel or rSm-TSP-2/Alhydrogel+AP10-701 or Engerix-B | Recruiting | NCT03910972 [75] |
| **Antigen:** rSm-TSP-2  **Adjuvant:** Aldydrogel, AP10-701 | **Experimental model:** humans (healthy male and non-pregnant female adults 18-50 years from *S. mansoni* endemic area Minas Gerais, Brazil)  **Administration:** immunization: i.m.  *Phase Ib trial on safety, reactogenicity, and immunogenicity of Sm-TSP-2/Alhydrogel®* *with/without AP 10-701 among healthy exposed adults*  **Immunization VG1:** 10ug rSm-TSP-2/Alhydrogel or 10ug rSm-TSP-2/Alhydrogel+AP10-701 or EuvaxB followed by 2 boosters of 10ug rSm-TSP-2/Alhydrogel or 10ug rSm-TSP-2/Alhydrogel+ AP10-701 or EuvaxB each on day 57 and 113  **Immunization VG2:** 30ug rSm-TSP-2/Alhydrogel or 30ug rSm-TSP-2/Alhydrogel+ AP10-701 or EuvaxB followed by 2 boosters of 30ug rSm-TSP-2/Alhydrogel or 30ug rSm-TSP-2/Alhydrogel+ AP10-701 or EuvaxB each on day 57 and 113  **Immunization VG3:** 100ug rSm-TSP-2/Alhydrogel or 100ug rSm-TSP-2/Alhydrogel+ AP10-701 or EuvaxB followed by 2 boosters of 100ug rSm-TSP-2/Alhydrogel or 100ug rSm-TSP-2/Alhydrogel+ AP10-701 or EuvaxB each on day 57 and 113 | Completed, not yet published | NCT03110757 [74] |
| **Antigen:** rSm-TSP-2 (expression system: *P. pastoris*)  **Adjuvant:** Aldydrogel, GLA-AF | **Experimental model:** humans (healthy male and non-pregnant female American adults 18-50 years from *S. mansoni* non-endemic area)  **Administration:** immunization: i.m.  *Phase I trial on safety, reactogenicity, and immunogenicity of Sm-TSP-2/Alhydrogel®* *with/without GLA-AF among healthy non-exposed adults*  **Immunization VG1:** 10ug rSm-TSP-2/Alhydrogel or 10ug rSm-TSP-2/Alhydrogel+GLA-AF or placebo (normal saline) followed by 2 boosters of 10ug rSm-TSP-2/Alhydrogel or 10ug rSm-TSP-2/Alhydrogel+GLA-AF or placebo each on day 57 and 113; follow-up for 12 months after 2^nd^ booster  **Immunization VG2:** 30ug rSm-TSP-2/Alhydrogel or 30ug rSm-TSP-2/Alhydrogel+GLA-AF or placebo (normal saline) followed by 2 boosters of 30ug rSm-TSP-2/Alhydrogel or 30ug rSm-TSP-2/Alhydrogel+GLA-AF or placebo each on day 57 and 113; follow-up for 12 months after 2^nd^ booster  **Immunization VG3:** 100ug rSm-TSP-2/Alhydrogel or 100ug rSm-TSP-2/Alhydrogel+GLA-AF or placebo (normal saline) followed by 2 boosters of 100ug rSm-TSP-2/Alhydrogel or 100ug rSm-TSP-2/Alhydrogel+GLA-AF or placebo each on day 57 and 113; follow-up for 12 months after 2^nd^ booster | **Participation:** 100% 1^st^ dose, 93% 2^nd^ dose, 85% 3^rd^ dose; 11 subjects dropped out  **Safety:** no SAEs; 17 subjects with mild clinical laboratory abnormalities e.g. changes in platelet counts or serum creatinine levels; AE e.g. 37 subjects with headache and fatigue, 57 subjects with tenderness and pain, 46 subjects with itching and mild decreased heart rate  **Immunogenicity:** IgG peak on day 127 in VGs2-3, similar level at all time points in VG1, decrease by day 293 in VGs1-3; no IgG in placebo recipients | Keitel WA,  et al. 2019; NCT02337855 [50,73] |
| **Antigen:** pUMVC3/ rSm29, pUMVC3/ rSmTSP-2, pUMVC3/rSM29 N/C-terminus/rSmTSP-2 (expression system: *E. coli* BL21, BHK-21 cells) | **Experimental model:** C57BL/6 mice  **Administration:** immunization: i.m.; challenge: p.c.  **Immunization VG1:** 100ug pUMVC3/rSm29 followed by 3 boosters of 100ug pUMVC3/rSm29 each on day 15, 30 and 45 (CG: 100ug pUMVC3); challenge with 100 *S. mansoni* cercariae (LE strain) on day 60; death on day 105  **Immunization VG2:** 100ug pUMVC3/rSmTSP-2 followed by 3 boosters of 100ug pUMVC3/rSmTSP-2 each on day 15, 30 and 45 (CG: 100ug pUMVC3); challenge with 100 *S. mansoni* cercariae (LE strain) on day 60; death on day 105  **Immunization VG3:** 100ug pUMVC3/rSM29 N/C-terminus/rSmTSP-2 followed by 3 boosters of 100ug pUMVC3/rSM29 N/C-terminus/rSmTSP-2 each on day 15, 30 and 45 (CG: 100ug pUMVC3); challenge with 100 *S. mansoni* cercariae (LE strain) on day 60; death on day 105  **Immunization VG4:** 50ug pUMVC3/rSm29 + 50ug pUMVC3/rSmTSP-2 followed by 3 boosters of 50ug pUMVC3/rSm29 + 50ug pUMVC3/rSmTSP-2 each on day 15, 30 and 45 (CG: 100ug pUMVC3); challenge with 100 *S. mansoni* cercariae (LE strain) on day 60; death on day 105 | **Worm reduction:** 17-22% in VG1; 22% in VG2; 31-32% in VG3; 24-32% in VG4  **Granuloma reduction** (hepatic)**:** 28% in VG1; 30% in VG2; 37% in VG3; 26% in VG4  **Immunogenicity:** total IgG highest in VG1 and VG3; splenocytes proliferated for IFN-𝛾 and TNF-𝛼 in VGs1-4 depending on stimulus | Gonçalves de Assis NR,  et al. 2015 [71] |
| **Antigen:** rSmTSP-2/Sm29 N-terminus (chimera A); rSmTSP-2/Sm29 C-terminus (chimera-B)  **Adjuvant:** CpG-alum | **Experimental model:** C57BL/6 mice  **Administration:** immunization: s.c.; challenge: p.c.  **Immunization VG1:** 25ug rSmTSP-2/Sm29 N-terminus+20ug CpG-alum followed by 2 boosters of 25ug rSmTSP-2/Sm29 N-terminus+20ug CpG-alum each on day 15 and 30 (CG: 20ug CpG-alum); challenge with 100 *S. mansoni* cercariae (LE strain) on day 45; death on day 90  **Immunization VG2:** 25ug rSmTSP-2/Sm29 C-terminus+20ug CpG-alum followed by 2 boosters of 25ug rSmTSP-2/Sm29 C-terminus+20ug CpG-alum each on day 15 and 30 (CG: 20ug CpG-alum); challenge with 100 *S. mansoni* cercariae (LE strain) on day 45; death on day 90  **Immunization VG3:** 25ug Sm29+20ug CpG-alum followed by 2 boosters of 25ug Sm29+20ug CpG-alum each on day 15 and 30 (CG: 20ug CpG-alum); challenge with 100 *S. mansoni* cercariae (LE strain) on day 45; death on day 90 | **Worm reduction:** 28% in VG1; 35% in VG2; 20% in VG3  **Granuloma reduction** (hepatic)**:** 48% in VG1; 31% in VG2; 38% in VG3  **Immunogenicity:** total IgG, IgG1, and IgG2a in VGs1-3; highest titers for total IgG and IgG2a in VG2; splenocytes proliferated for IFN-𝛾 and TNF-𝛼 in VGs1-2 | Pinheiro CS,  et al. 2014 [64] |
| **Antigen:** Sm-TSP-2/5B, Sm-TSP-2 (expression system: *E. coli* Rosetta-Gami, E. coli BL21-Al  **Adjuvant:** CpG-alum | **Experimental model:** C57BL/6 mice  **Administration:** immunization: i.p.; challenge: s.c.  **Immunization VG1:** 25ug Sm-TSP-2+5ugCpG-alum13mg/ml followed by 2 boosters of 25ug Sm-TSP-2+5ugCpG-alum13mg/ml each on day 14 and 28 (CG: MBP); challenge with 120 *S. mansoni* cercariae on day 42; death on day 91  **Immunization VG2:** 25ug Sm-TSP-2/5B+5ugCpG-alum13mg/ml followed by 2 boosters of 25ug Sm-TSP-2/5B+5ugCpG-alum13mg/ml each on day 14 and 28 (CG: MBP); challenge with 120 *S. mansoni* cercariae on day 42 | **Worm reduction:** 25-27% in VG1; 54-58% in VG2  **Egg reduction** (hepatic**):** 20-27% in VG1; 48-56% in VG2  **Immunogenicity:** anti-Sm-TSP-2 total IgG, IgG1 and weak IgG2A in VGs1-2, but higher titers in VG2; splenocytes proliferated for IFN-𝛾, IL-4 and IL-10 in VGs1-2 | Pearson MS,  et al. 2012 [70] |
| **Antigen:** rSm-TSP-1, rSm-TSP-2 (expression system: *E. coli*)  **Adjuvant:** CFA, IFA | **Experimental model:** CBA/CaH mice  **Administration:** challenge: s.c.  **Immunization:** 25ug rSm-TSP1/25ug rSm-TSP2+CFA followed by 2 boosters of 25ug rSm-TSP1/25ug rSm-TSP2+IFA each on day 14 and 28 (CG: 25ug *E. coli* r-thioredoxin+CFA/IFA); challenge with 120 *S. mansoni* cercariae on day 42; death on day 84 | **Worm reduction:** 57% for TSP-1; 34% for TSP-2  **Egg reduction:** 52% hepatic and 69% fecal for TSP-1; 64% hepatic and 65% fecal for TSP-2  **Immunogenicity:** IgG1 and IgG2A against TSP-1 and TSP-2 in mice; higher anti-TSP-2 IgG1 and IgG3 in putative resistant than chronically infected humans and no antibody response to TSP-1; all IgG subclasses and IgE against soluble egg and worm antigens in chronically infected | Tran MH,  et al. 2006 [66] |

Abbreviations: TSP=tetraspanin; VG=vaccine group; CG=control group; s.c.=subcutaneous; i.m.=intramuscular; i.p.=intraperitoneal; p.c.=percutaneous; CFA=complete Freund’s adjuvant; IFA=incomplete Freund’s adjuvant; CpG-alum=unmethylated CpG dinucleotides with aluminum hydroxide; MBP=maltose binding protein; BHK-21 cells=hamster kidney cells; IL=interleukin; Ig=immunoglobulin; IFN-𝛾=interferon gamma; TNF-𝛼=tumor necrosis factor alpha; Alhydrogel=aluminum hydroxide salt; GLA-AF=glucopyranosyl lipid A in aqueous formulation; AP10-701=glucopyranosyl lipid A (aqueous formulation); EuvaxB/Engerix-B=hepatitis B vaccine; S*. mansoni*/Sm=*Schistosoma mansoni*; *S. haematobium*=*Schistosoma haematobium*; *E. coli*=*Escherichia coli*; *P. pastoris*=*Pichia pastoris*; SAE=serious adverse event; AE=adverse event.

Note: References were obtained through systematic searches in PubMed without restrictions in language and time, including a reference search among the publications included, and at the U.S. National Library of Medicine for clinical trial; the last searches were performed on April 25, 2021.
